# Norepinephrinergic projection from locus coeruleus to parafascicular nucleus promotes pain and anxiety-like behaviors in mice

**DOI:** 10.1172/jci.insight.198224

**Published:** 2026-04-07

**Authors:** Zhong-Yi Liu, Fei Li, Li-Ming Liu, Yao-Hua Liu, Jia Li, Zi-Ang Li, Jin Cheng, Tian-Yu Zhao, Hui-Min Tian, Dong-Ning Li, Sha-Sha Tao, Hui Li, Fen-Sheng Huang, Yun-Qing Li

**Affiliations:** 1 Department of Anatomy and K.K. Leung Brain Research Centre, The Fourth Military Medical University, Xi’an, China.; 2 Department of Anatomy, Fujian Medical University, Fuzhou, China.; 3 Department of Anatomy, School of Medicine, Dali University, Dali, China.; 4 Department of Anatomy, Baotou Medical College, Inner Mongolia University of Science and Technology, Baotou, China.; 5 Department of Anatomy, Zunyi Medical University, Zunyi, China.; 6 Department of Anatomy, School of Medicine, Zhengzhou University, Zhengzhou, China.; 7 Institute of Neuroscience and Physiology, Göteborg University, Göteborg, Sweden.

**Keywords:** Cell biology, Neuroscience, Molecular biology, Pharmacogenetics, Pharmacology

## Abstract

Chronic neuropathic pain is frequently comorbid with anxiety disorders, yet the neural circuits underlying this interaction remain poorly defined. The parafascicular nucleus (PF) of the thalamus integrates nociceptive and affective signals, but its specific regulatory mechanisms in pain-anxiety comorbidity are not well known. Using spared nerve injury (SNI) model mice, we combined viral neural tracing, chemogenetics, pharmacology, and electrophysiology to dissect the locus coeruleus (LC)–PF neural pathway. Viral tracing revealed monosynaptic projections from norepinephrinergic (NEergic) neurons in the dorsal LC to Ca^2+^/calmodulin–dependent protein kinase IIα–immunopositive (CaMKIIα^+^) neurons within the PF. Chemogenetic inhibition and activation of this pathway were performed in naive and SNI mice, alongside intra-PF microinjection of the α-2 adrenergic receptor (ADRA2) antagonist yohimbine. Behavioral tests assessed mechanical/thermal hypersensitivity and anxiety-like behaviors. Results showed that 92.1% of PF-projecting LC neurons were NEergic, with 70.1% localized dorsally. Chemogenetic inhibition of the LC^NE^-PF^CaMKIIα^ neural pathway significantly alleviated both acute-phase mechanical hypersensitivity (<7 days after surgery) and chronic-phase anxiety-like behaviors in SNI mice, while activation of this pathway induced pain sensitization and anxiety-like behaviors in naive mice. Intra-PF yohimbine reversed SNI-induced allodynia and anxiety-like behaviors. Electrophysiology confirmed that yohimbine increased PF neuronal intrinsic excitability. These results suggest that the LC^NE^-PF^CaMKIIα^ neural pathway promotes neuropathic pain and comorbid anxiety via ADRA2-mediated suppression of PF neuronal activity. Targeted inhibition of this circuit may represent a therapeutic strategy for pain-related affective disorders.

## Abstract 2

The LC^NE^-PF^CaMKIIα^ pathway plays a role in the comorbidity of pain and anxiety by mediating the suppression of PF neurons via ADRA2.

## Graphical abstract

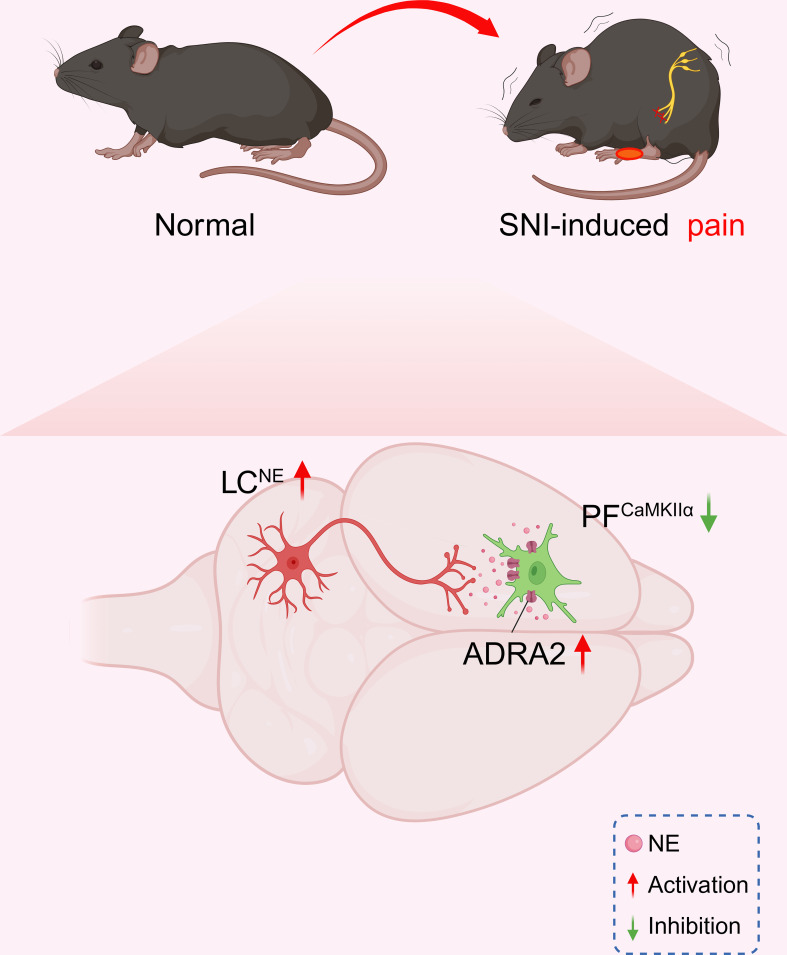

## Introduction

Chronic neuropathic pain affects over 20% of the global population and is frequently comorbid with anxiety disorders, severely impairing quality of life and treatment outcomes (1, 2). Despite substantial clinical evidence, the neural circuit mechanisms underlying this comorbidity remain poorly defined, limiting targeted therapeutic strategies. The parafascicular nucleus (PF) of the thalamus serves as a critical hub integrating nociceptive and affective signals (3). It receives inputs from pain-processing regions (e.g., amygdala) and projects to cortical areas involved in emotional regulation (e.g., anterior cingulate cortex) (4). Rodent studies demonstrate that PF neurons are activated during nociception (5), and play a critical role in regulating negative emotions such as anxiety (6). Notably, the PF expresses high densities of adrenergic receptors (ARs) (7), suggesting modulation by norepinephrine (NE) — a key neuromodulator in stress and pain pathways. However, the source of NEergic inputs to the PF and their functional role in pain-anxiety comorbidity are unknown.

The locus coeruleus (LC), the brain’s primary NEergic nucleus, regulates both nociception and affective states via divergent projections (8). While the LC-spinal pathway suppresses pain by activating α-2 adrenergic receptor (ADRA2) (9), LC-cortical projections (e.g., to the prefrontal cortex) promote pain and anxiety (10). Interestingly, macaque tract-tracing identified direct LC-PF connections (11), and electrophysiological studies suggest α-ARs modulate PF nociceptive responses (12). Yet, whether this pathway drives pain-anxiety comorbidity, and through which molecular mechanisms, remains unexplored.

Here, we hypothesized that hyperactivity of LC-derived NEergic projections to Ca^2+^/calmodulin–dependent protein kinase IIα–immunopositive (CaMKIIα^+^) neurons in the PF promotes neuropathic pain and comorbid anxiety via ADRA2 signaling. We used the spared nerve injury (SNI) model to anatomically define the monosynaptic LC^NE^-PF^CaMKIIα^ projection through viral tracing, and functionally dissected this pathway using chemogenetics and pharmacology, and further elucidated the ADRA2-mediated inhibition of PF neuron intrinsic excitability through electrophysiology. Our findings reveal a circuit mechanism underlying pain-anxiety comorbidity and identify ADRA2 in the PF as a potential therapeutic target.

## Results

### Activation of CaMKIIα^+^ neurons in the PF generates analgesic and anxiolytic effects.

Previous studies have reported that rodents develop immediate sensory hypersensitivity after nerve injury, followed by anxiety, depression, and cognitive dysfunction within weeks (typically 3–8 weeks, depending on species and animal models) (13, 14). In this study, we assessed the impact of neuropathic pain on pain thresholds and anxiety-like behaviors in mice using the SNI model (Supplemental Figure 1A; supplemental material available online with this article; https://doi.org/10.1172/jci.insight.198224DS1). Mechanical pain thresholds were measured preoperatively and on postoperative days 1, 4, 7, 10, 13, 16, 19, 21, and 28. The results revealed that compared with the Sham group, mice in the SNI group developed mechanical hypersensitivity in both ipsilateral and contralateral sides to the injury on postoperative day 1, with the hind paw withdrawal threshold (PWT) significantly decreased, reaching its nadir on day 4 and persisting through day 28 (Supplemental Figure 1B). In the thermal hyperalgesia test, SNI mice exhibited significant pain sensitization to 52°C and 56°C hot plate stimuli, showing significantly shortened hind paw withdrawal latency (PWL), while no significant differences were detected at 48°C (Supplemental Figure 1C). Anxiety-like behaviors were assessed 5 weeks after surgery. The open field test (OFT) revealed significantly reduced time in center of SNI mice, with no differences in total distance (Supplemental Figure 1, D and E). In the elevated plus maze (EPM), SNI mice exhibited significant decreases in both the number of entries into open arms and the time spent in open arms (Supplemental Figure 1, F and G). These results indicate that neuropathic pain induces persistent pain hypersensitivity and anxiety-like behaviors in mice without affecting locomotor activity.

**Figure 1:**
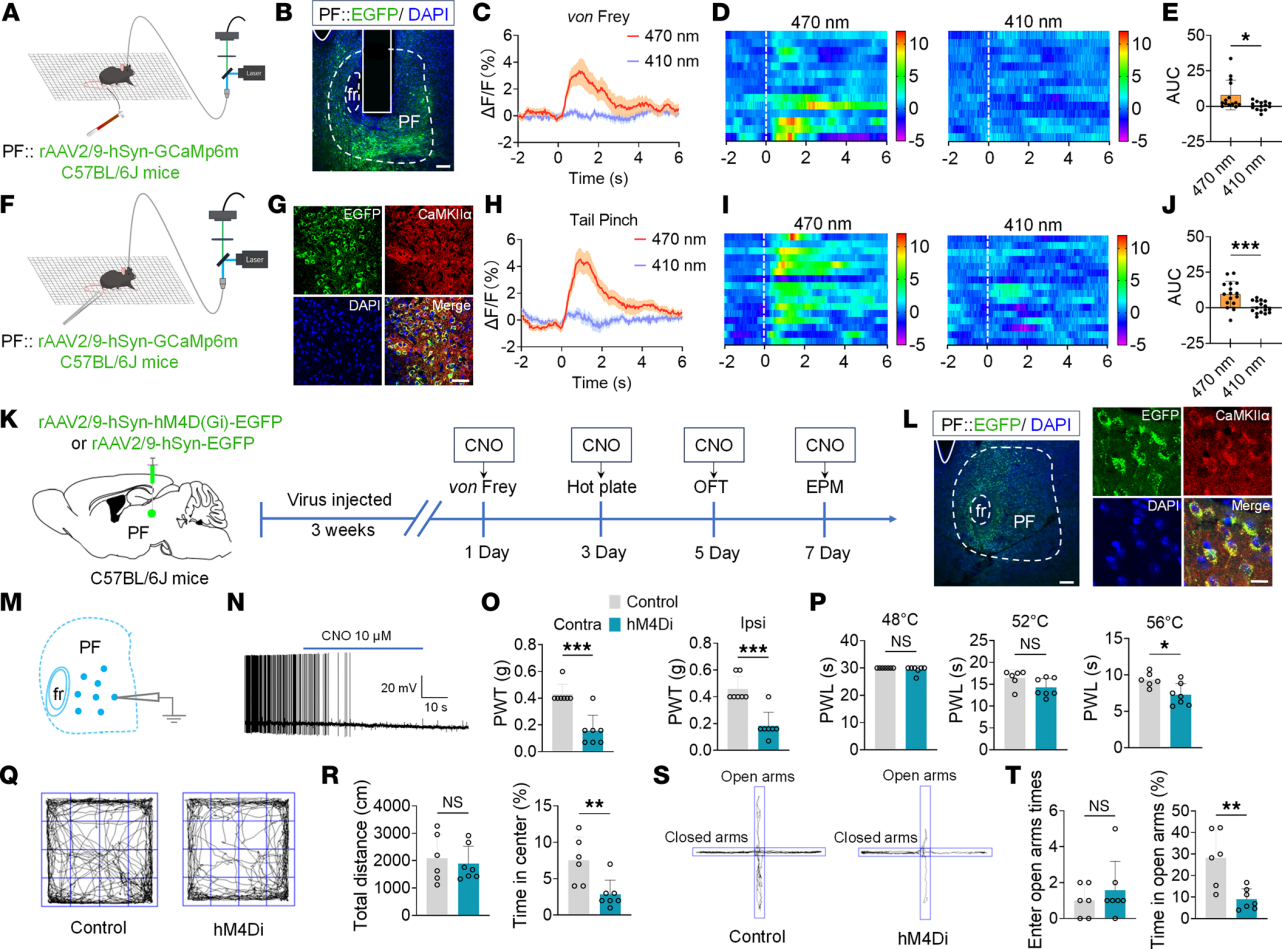
Nociceptive stimuli activate PF^CaMKIIα^ neurons in mice. (**A** and **F**) Schematic of von Frey (**A**) and tail pinch stimulus (**F**) paradigms using fiber photometry. (**B** and **G**) Representative images of viral injection sites (green) in the PF (**B**) and immunofluorescence colocalization of GCaMP6m^+^ (green) with CaMKIIα^+^ neurons (red) (**G**). (**C**–**E**) Ca²^+^ signal dynamics in PF^CaMKIIα^ neurons during von Frey stimulation: corrected ΔF/F mean values (470 nm vs. 410 nm) (**C**), heatmaps (**D**), and quantified area under the curve (AUC) (**E**). (**H**–**J**) Ca²^+^ signal dynamics in PF^CaMKIIα^ neurons during tail pinch stimulation: ΔF/F mean values (**H**), heatmaps (**I**), and quantified AUC (**J**). (**K**) Schematic of experimental design. (**L**) Representative images of viral injection sites (green) in the PF (left) and colocalization of EGFP^+^ (green) with CaMKIIα^+^ neurons (red) (right). (**M**) Schematic of patch-clamp experimental design. (**N**) Representative current-clamp recordings of hM4Di neurons treated with 10 μM CNO. (**O** and **P**) Effects of chemogenetic inhibition of the PF^CaMKIIα^ neurons on mechanical (**O**) and thermal (**P**) pain thresholds in naive mice. (**R**) Total distance (left) and percentage time in center zone (right) during OFT in Control and hM4Di mice. (**T**) Times of open arm entries (left) and percentage time in open arms (right) during EPM in Control and hM4Di mice. (**Q** and **S**) Representative OFT (**Q**) and EPM (**S**) movement trajectories for Control and hM4Di mice. Scale bars: 120 μm (**B** and left panel of **L**), 20 μm (**G**), 8 μm (right panel of **L**). All data are expressed as mean ± SEM (**E**, *n* = 14; **J**, *n* = 16; **O**–**T**, *n* = 7). Statistical analysis: 2-tailed unpaired *t* test. *P < 0.05, **P < 0.01, ***P < 0.001. NS, not significant.

To investigate whether PF neurons respond to nociceptive stimuli, fiber photometry was used to record Ca^2+^ activity in PF neurons of mice during von Frey filament and tail pinch stimulation (Figure 1, A and F). Three weeks after viral expression, immunofluorescence histochemistry confirmed extensive colocalization of genetically encoded calcium indicator protein 6 medium (GCaMP6m) and Ca^2+^/calmodulin–dependent protein kinase IIα (CaMKIIα) in PF neurons (Figure 1, B and G), validating that the recorded Ca^2+^ signals originated specifically from PF^CaMKIIα^ neurons. Fiber photometry results demonstrated that both von Frey filament and tail pinch stimuli elicited a significant increase in Ca^2+^-dependent fluorescence intensity (ΔF/F ratio) at an excitation wavelength of 470 nm compared with the reference wavelength of 410 nm in PF^CaMKIIα^ neurons (Figure 1, C–E and H–J). These findings indicate that nociceptive stimuli can activate CaMKIIα^+^ neurons in the PF.

To investigate the function of PF^CaMKIIα^ neurons in pain and anxiety-like behaviors in mice, we employed chemogenetic techniques to inhibit these neurons in normal mice (Figure 1K). Electrophysiological recordings revealed membrane hyperpolarization and markedly reduced spontaneous firing rates in hM4Di-expressing neurons following clozapine *N*-oxide (CNO) administration (Figure 1, M and N), confirming successful viral infection. Immunofluorescence histochemistry revealed colocalization of enhanced green fluorescent protein (EGFP) and CaMKIIα^+^ neurons in PF (Figure 1L). Behavioral results showed that the hM4Di group exhibited significantly reduced mechanical PWT and thermal PWL (Figure 1, O and P). In the OFT and EPM, the hM4Di group showed no significant differences in total distance or open arm entries compared to the control group, but displayed significantly decreased time spent in the center zone and open arms (Figure 1, Q–T). Conversely, chemogenetic activation of PF^CaMKIIα^ neurons showed no significant modulation of nociceptive thresholds or anxiety-related behaviors, although hM3Dq-expressing neurons exhibited markedly increased spontaneous firing frequencies after CNO administration (Supplemental Figure 2, A–I).

**Figure 2:**
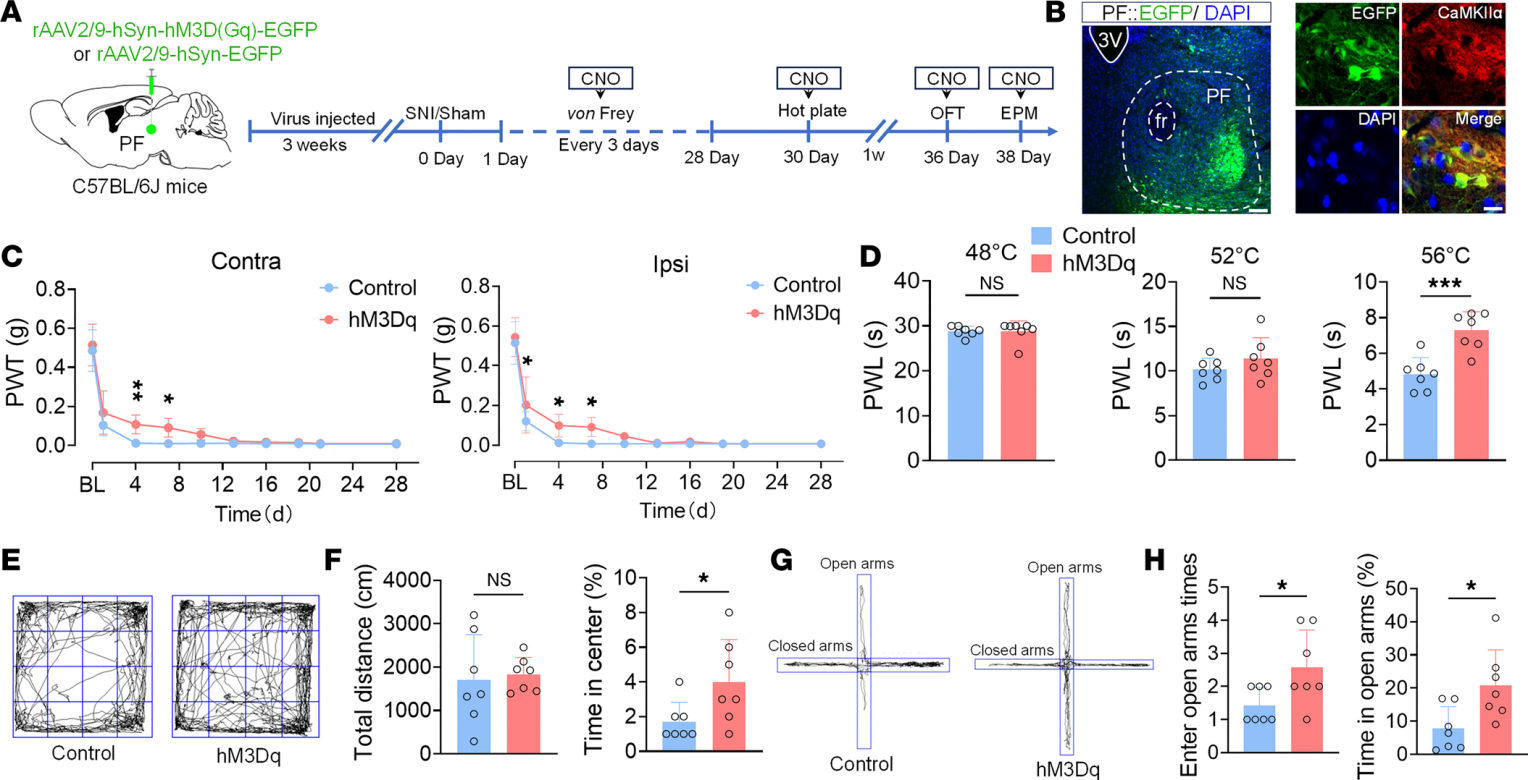
Activation of PF^CaMKIIα^ neurons alleviates pain and anxiety-like behaviors in SNI mice. (**A**) Schematic of experimental design. (**B**) Representative images of viral injection sites (green) in the PF (left) and colocalization of EGFP^+^ (green) with CaMKIIα^+^ neurons (red) (right). Scale bars: 120 μm (left) and 8 μm (right). (**C**) PWT (grams) in ipsilateral (right) and contralateral (left) hind paws of Control and hM3Dq mice on postoperative days 0, 1, 4, 7, 10, 13, 16, 19, 21, and 28. (**D**) PWL (seconds) of Control and hM3Dq mice exposed to 48°C, 52°C, and 56°C hot plates. (**E**) Representative OFT movement trajectories of Control and hM3Dq mice. (**F**) Total distance (left) and percentage time in center zone (right) during OFT in Control and hM3Dq mice. (**G**) Representative EPM movement trajectories of Control and hM3Dq mice. (**H**) Times of open arm entries (left) and percentage time in open arms (right) during EPM in Control and hM3Dq mice. 3V, third ventricle. All data are expressed as mean ± SEM (*n* = 7). Statistical analysis: 2-way ANOVA (**C**); 2-tailed unpaired *t* test (**D**, **F**, and **H**). *P < 0.05, **P < 0.01, ***P < 0.001. NS, not significant.

To investigate the role of PF^CaMKIIα^ neurons under pathological conditions, chemogenetic viruses were injected into naive mice, and the SNI model was established subsequently (Figure 2, A and B). Behavioral results demonstrated a significant increase in the mechanical PWT of SNI mice within 7 days postoperatively, although thresholds progressively declined after 7 days (Figure 2C), while thermal hyperalgesia was significantly alleviated on postoperative day 28 (Figure 2D). In the OFT and EPM, the hM3Dq mice showed significant amelioration of anxiety-like behaviors (Figure 2, E–H). Subsequent inhibition of PF^CaMKIIα^ neurons in SNI mice (Supplemental Figure 3A) revealed no significant changes in mechanical/thermal pain thresholds or anxiety-like behaviors (Supplemental Figure 3, B–G). This phenomenon may result from the peak effects of SNI-induced allodynia and anxiety, suggesting that enhanced activity of PF^CaMKIIα^ neurons might be a prerequisite for alleviating pain and anxiety.

### The PF receives direct inputs from NEergic neurons of the LC.

**Figure 3:**
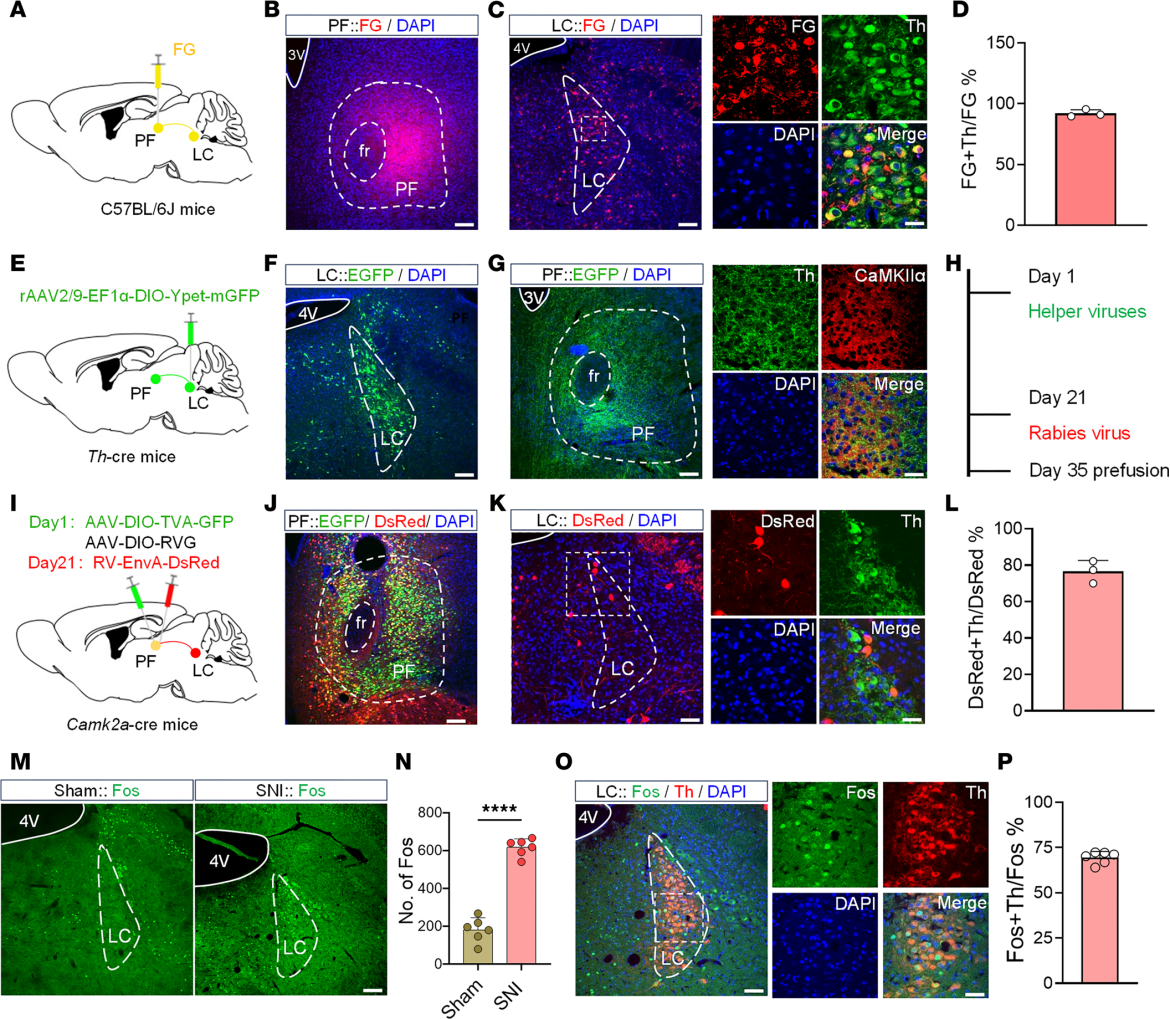
Monosynaptic NEergic projection from the LC to the PF^CaMKIIα^. (**A**) Schematic of Fluoro-Gold (FG) injection into the PF. (**B**) Representative image of FG injection site in the PF. (**C**) Representative images of FG^+^ neurons (red, left) colocalization with Th^+^ neurons (green, right) in the LC. (**D**) Percentage of FG^+^ and Th^+^ double-labeled (FG^+^Th^+^) neurons among FG^+^ neurons in the LC (*n* = 3). (**E**) Schematic of anterograde virus injection into the LC. (**F**) Representative image of viral injection site in the LC. (**G**) Representative images of EGFP-labeled fibers/terminals (green, left) in close contact with CaMKIIα^+^ neurons (red) in the PF (right). (**H** and **I**) Timeline and schematic for virus injection of retrograde trans-monosynaptic tracing strategy. (**J**) Representative image of viral injection site in the PF. (**K**) Representative images of DsRed^+^ neurons (red, left) colocalization with Th^+^ neurons (green) in the LC (right). (**L**) Percentage of DsRed^+^Th^+^ neurons among DsRed^+^ neurons in the LC (*n* = 3). (**M**) Representative images of Fos expression in the LC of Sham and SNI mice. (**N**) The number of Fos^+^ neurons in the LC of Sham and SNI mice (*n* = 6). (**O**) Representative images of Fos^+^ neurons (green) colocalization with Th^+^ neurons (red, left), and their high-magnification images (right). (**P**) Percentage of Fos^+^Th^+^ neurons among Fos^+^ neurons in SNI mice (*n* = 6). Scale bars: 120 μm (**B**, **F**, **J**, and **M** and the left panel of **C**, **G**, **K**, and **O**) and 20 μm (the right panel of **C**, **G**, **K**, and **O**). 4V, fourth ventricle. All data presented as mean ± SEM. Statistical analysis: 2-tailed unpaired *t* test (**N**). ****P < 0.0001.

Based on these findings, we further explored the upstream pain-related brain regions of the PF to identify functional connections integrating pain and anxiety. The retrograde tracer Fluoro-Gold (FG) was injected into the PF (Figure 3, A and B), and dense FG retrograde–labeled (FG^+^) neurons were observed in the LC (Figure 3C). Immunofluorescence histochemical staining revealed that 92.1% of FG^+^ neurons colocalized with tyrosine hydroxylase–immunopositive (Th^+^) neurons in the LC (Figure 3, C and D, and Supplemental Table 1), with 70.1% concentrated in the dorsal region (Supplemental Figure 4 and Supplemental Table 1), suggesting direct projections from LC^NE^ neurons to the PF. To validate this pathway, anterograde tracers were injected into the LC of *Th*-cre mice (Figure 3, E and F). Virus-labeled fibers and terminals were observed in the PF closely in contact with CaMKIIα^+^ neurons (Figure 3G), further confirming NEergic neuron projections from the LC to PF^CaMKIIα^ neurons. Retrograde monosynaptic tracing was performed by injecting rabies virus into the PF of *Camk2a*-cre mice (Figure 3, H–J). Results showed that 76.47% of DsRed^+^ neurons in the LC colocalized with Th^+^ neurons (Figure 3, K and L, and Supplemental Table 2). These tracing experiments confirmed that NEergic neurons in the LC project to CaMKIIα^+^ neurons in the PF. Next, we detected the Fos protein, a neuronal activity marker, in the LC of SNI mice. Results showed a significant increase in Fos^+^ neurons in the SNI group (182 ± 58.58 vs. 617 ± 41.39; Figure 3, M and N, and Supplemental Table 3), with 69.45% of Fos^+^ neurons colocalized with Th^+^ neurons (Figure 3, O and P, and Supplemental Table 4), indicating chronic pain activates LC^NE^ neurons.

### Activation of the LC^NE^-PF^CaMKIIα^ neural pathway induces nociceptive hypersensitivity and anxiety-like behaviors in naive mice.

**Figure 4:**
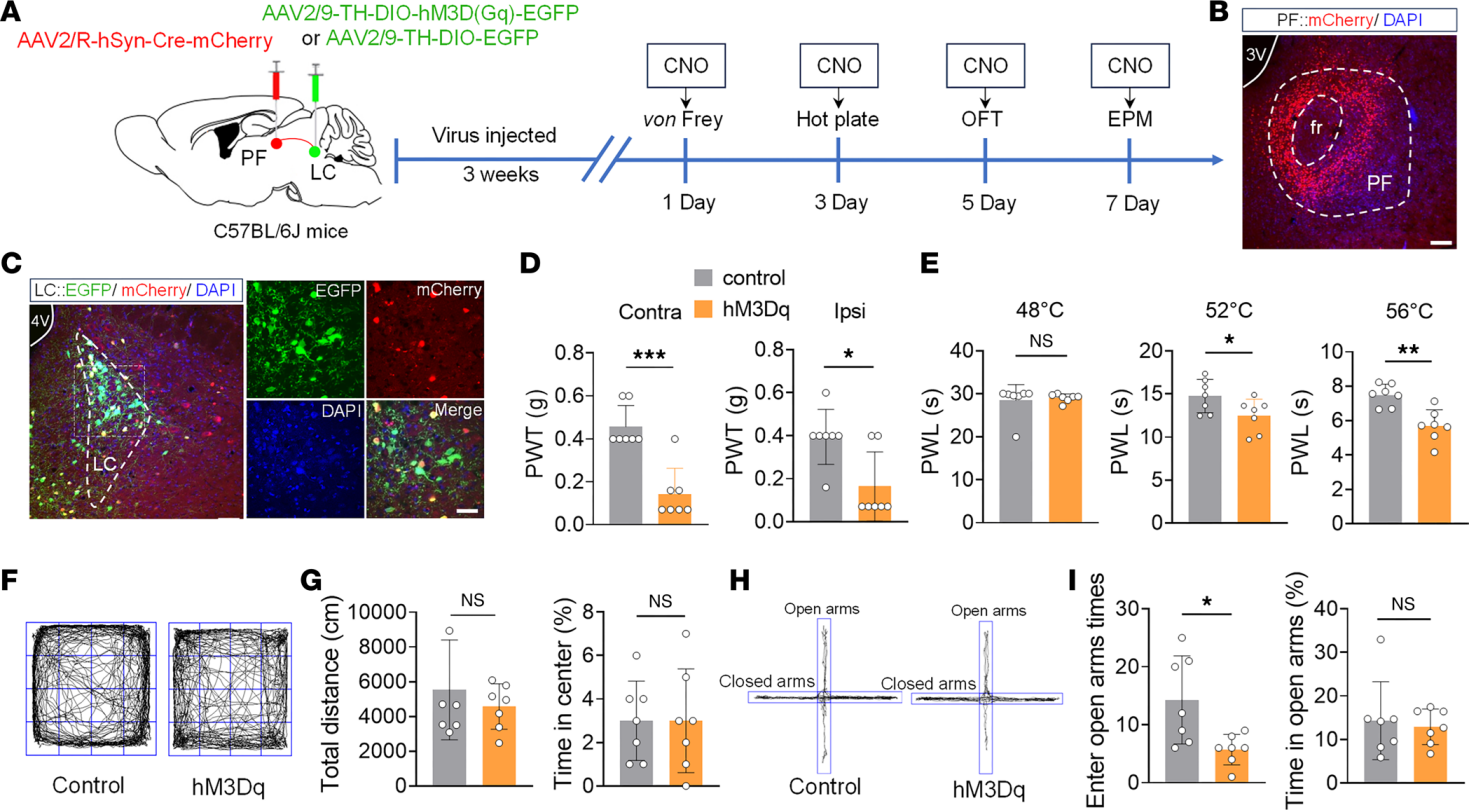
Activation of the LC^NE^-PF^CaMKIIα^ neural pathway promotes nociceptive hypersensitivity and induces anxiety-like behaviors. (**A**) Schematic of pathway activation experimental design. (**B**) Representative image of FG injection sites in the PF. (**C**) Representative images of EGFP-labeled neurons (green) colocalization with retrograde-labeled neurons (red) from the PF (left), and their high-magnification images (right). (**D** and **E**) Effects of chemogenetic activation of the LC^NE^-PF^CaMKIIα^ neural pathway on mechanical (**D**) and thermal (**E**) pain thresholds in naive mice. (**F**) Representative OFT movement trajectories for Control and hM3Dq mice. (**G**) Total distance (left) and percentage time in center zone (right) during OFT in Control and hM3Dq mice. (**H**) Representative EPM movement trajectories for Control and hM3Dq mice. (**I**) Times of open arm entries (left) and percentage time in open arms (right) during EPM in Control and hM3Dq mice. Scale bars: 120 μm (**B** and the left panel of **C**) and 20 μm (the right panel of **C**). All data presented as mean ± SEM (*n* = 7). Statistical analysis: 2-tailed unpaired *t* test (**D**, **E**, **G**, and **I**). *P < 0.05, **P < 0.01, ***P < 0.001. NS, not significant.

To investigate the role of the LC^NE^-PF^CaMKIIα^ neural pathway in pain and anxiety-like behaviors, we injected chemogenetic activation viruses into the LC and PF of C57BL/6J mice (Figure 4, A–C). Results showed that both PWT and PWL in hM3Dq group mice were significantly decreased (Figure 4, D and E), and anxiety-like behaviors were observed (Figure 4, F–I). Subsequent inhibition of the LC^NE^-PF^CaMKIIα^ neural pathway (Supplemental Figure 5A) revealed no statistically significant differences in mechanical/thermal pain thresholds or anxiety-like behaviors (Supplemental Figure 5, B–G). These findings suggest that activation of the LC^NE^-PF^CaMKIIα^ neural pathway under physiological conditions induces nociceptive sensitization and anxiety-like behaviors, while inhibition produces no significant behavioral effects.

### Chemogenetic inhibition of the LC^NE^-PF^CaMKIIα^ neural pathway alleviates pain and anxiety-like behaviors.

**Figure 5:**
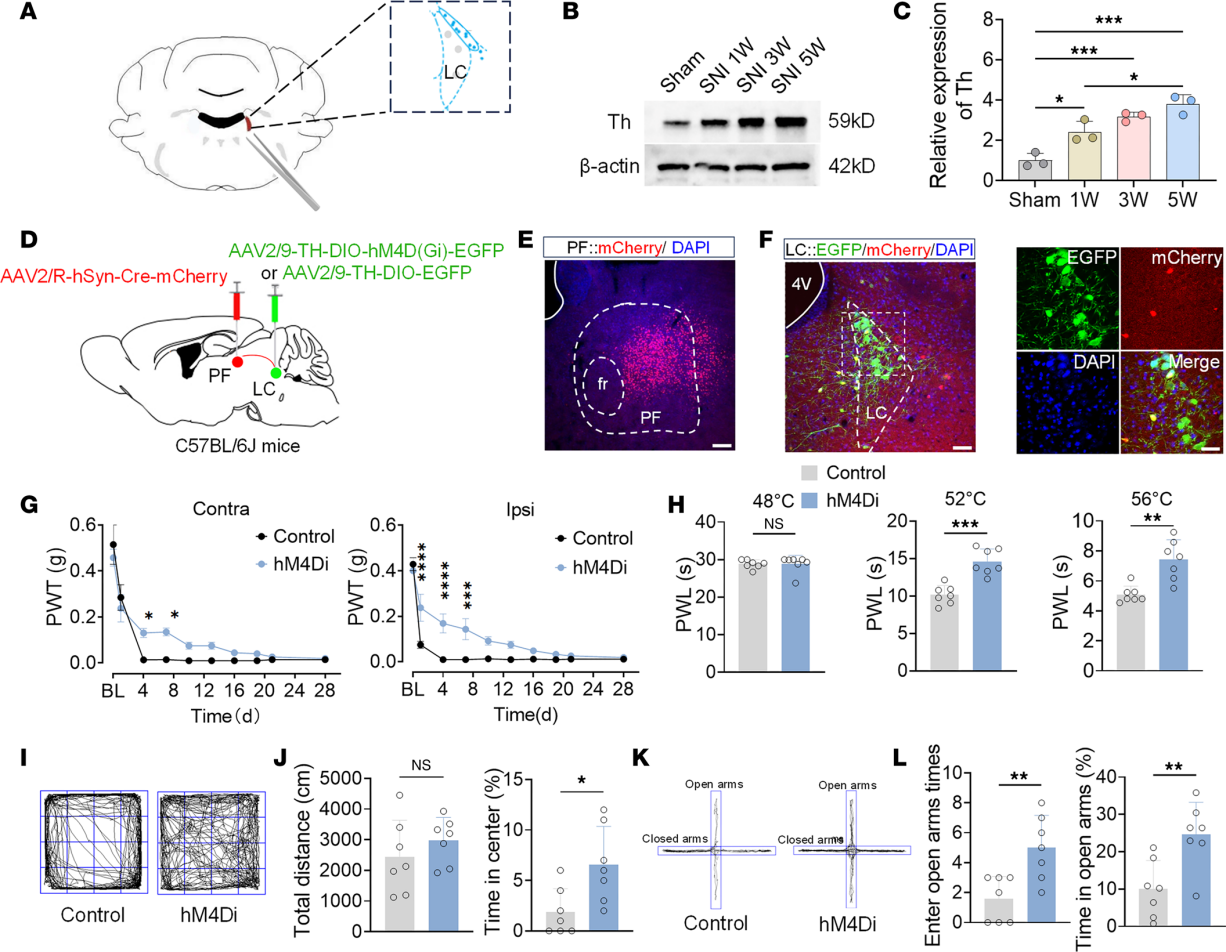
Inhibition of the LC^NE^-PF^CaMKIIα^ neural pathway alleviates pain and anxiety-like behaviors in SNI mice. (**A**) Schematic diagram of tissue sampling sites for Western blotting analysis. (**B**) Representative Western blotting (of 3 independent experiments) showing Th protein levels in the LC at different post-SNI time points. (**C**) Summarized data of Th protein expression levels. (**D**) Schematic of pathway inhibition experimental design. (**E**) Representative image of viral injection sites in the PF. (**F**) Representative image of EGFP-labeled neurons (green) colocalization with retrograde-labeled neurons (red) from the PF (left), and their high-magnification images (right). (**G**) PWT (grams) of ipsilateral (right) and contralateral (left) hind paws of Control and hM4Di mice on postoperative days 0, 1, 4, 7, 10, 13, 16, 19, 21, and 28. (**H**) PWL (seconds) of Control and hM4Di mice exposed to 48°C, 52°C, and 56°C hot plates. (**I**) Representative OFT movement trajectories. (**J**) Total distance (left) and percentage time in center zone (right) during OFT in Control and hM4Di mice. (**K**) Representative EPM movement trajectories. (**L**) Times of open arm entries (left) and percentage time in open arms (right) during EPM in Control and hM4Di mice. Scale bars: 120 μm (**E** and the left panel of **F**) and 20 μm (the right panel of **F**). All data presented as mean ± SEM (**C**, *n* = 3; **G**, **H**, **J**, and **K**, *n* = 7). Statistical analysis: 2-way ANOVA (**G**); 1-way ANOVA (**C**); or 2-tailed unpaired *t* test (**H**, **J**, and **L**). *P < 0.05; **P < 0.01; ***P < 0.001; ****P < 0.0001. NS, not significant.

Based on the above findings, we hypothesized that the inhibition of CaMKIIα^+^ neurons in the PF of SNI mice might be driven by NEergic inputs from the LC. To validate this hypothesis, semiquantitative Western blotting analysis of Th protein expression in the LC of Sham and SNI groups mice (postoperative days 7, 21, and 35) revealed progressive upregulation of Th in SNI mice (Figure 5, A–C), confirming sustained NE activation during chronic pain. To investigate the role of the LC^NE^-PF^CaMKIIα^ neural pathway in chronic pain, chemogenetic inhibition viruses were injected into the LC and PF of mice (Figure 5, D–F). Results demonstrated that hM4Di group mice exhibited significant alleviation of mechanical pain within 7 days after surgery (Figure 5G), although this effect gradually attenuated thereafter. By day 28, a marked improvement in thermal hyperalgesia was observed (Figure 5H), accompanied by concomitant amelioration of anxiety-like behaviors (Figure 5, I–L). Chemogenetic activation experiments (Supplemental Figure 6A) showed no statistically significant differences in pain thresholds or anxiety behaviors (Supplemental Figure 6, B–G), suggesting the pain and anxiety states in mice have reached pathological peak levels. These results indicate that inhibition of the LC^NE^-PF^CaMKIIα^ neural pathway alleviates chronic pain and anxiety-like behaviors, while activation has no effect.

### The LC-PF neural pathway mediates pronociceptive and anxiogenic effects via ADRA2 signaling in the PF.

**Figure 6:**
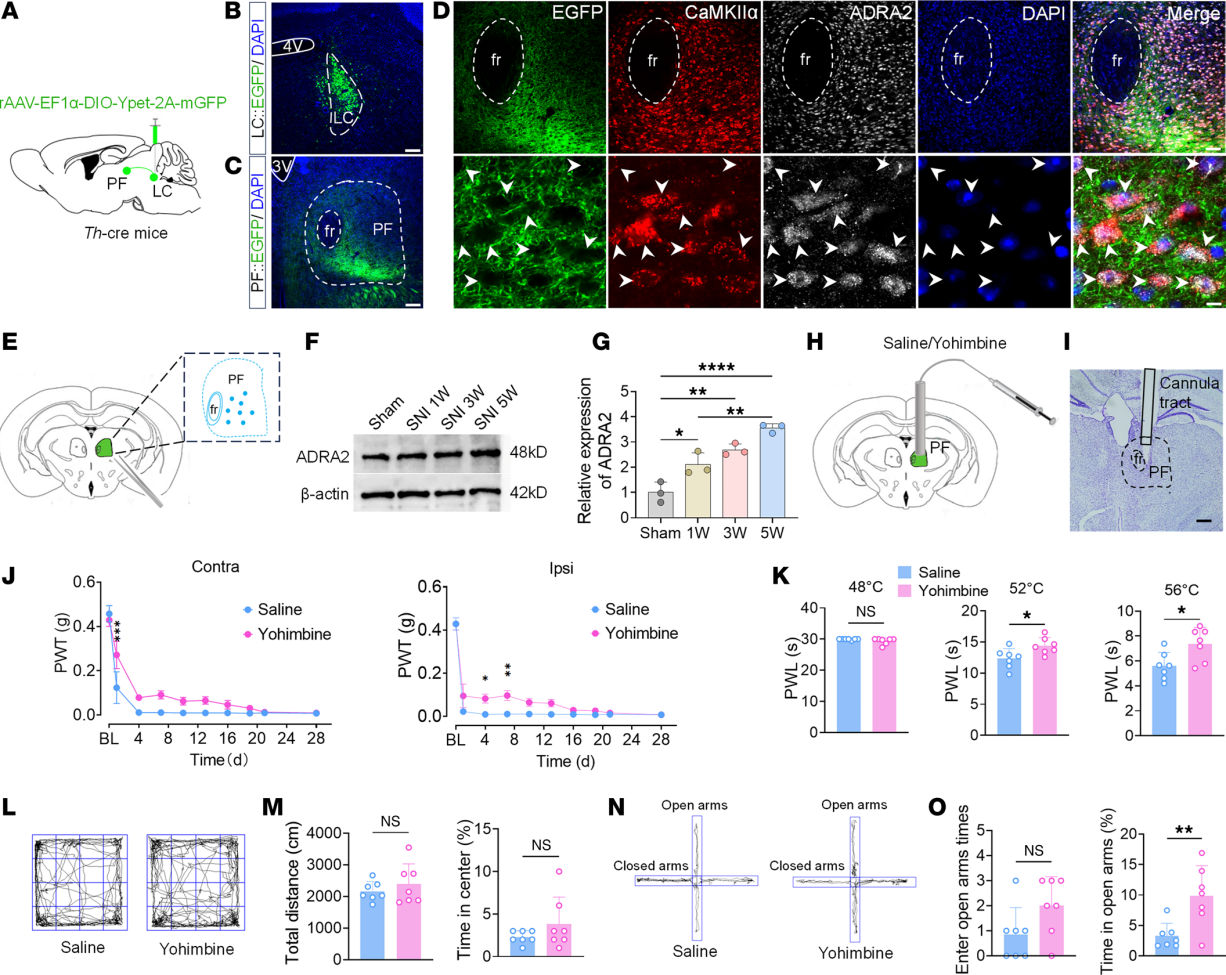
ADRA2 in the PF mediates chronic pain and anxiety. (**A**) Schematic of anterograde tracing virus injection into the LC. (**B**) Representative image of viral injection sites in the LC. (**C**) Representative image of anterograde virus-labeled fibers/terminals in the PF. (**D**) Representative images of close apposition of CaMKIIα^+^ neurons (red), ADRA2 (gray), and virus-labeled fibers/terminals (green). White arrows indicate quadruple-labeled neurons (CaMKIIα^+^ neurons, ADRA2^+^, virus-labeled fibers/terminals, and DAPI). (**E**) Schematic diagram of tissue sampling sites for Western blotting analysis. (**F**) Representative Western blotting (of 3 independent experiments) showing ADRA2 protein levels in the PF at different post-SNI time points. (**G**) Summarized data of ADRA2 protein expression levels. (**H**) Schematic of cannula implantation in the PF. (**I**) Representative image of cannula placement in the PF. (**J**) PWT (grams) in ipsilateral (right) and contralateral (left) hind paws of saline-treated and yohimbine-treated mice on postoperative days 0, 1, 4, 7, 10, 13, 16, 19, 21, and 28. (**K**) PWL (seconds) of saline-treated and yohimbine-treated mice exposed to 48°C, 52°C, and 56°C hot plates. (**L**) Representative OFT movement trajectories. (**M**) Total distance (left) and percentage time in center zone (right) during OFT in saline-treated and yohimbine-treated mice. (**N**) Representative EPM movement trajectories. (**O**) Times of open arm entries (left) and percentage time in open arms (right) during EPM in saline-treated and yohimbine-treated mice. Scale bars: 200 μm (**I**), 120 μm (**B** and **C**), 60 μm (the upper panel of **D**), and 8 μm (the lower panel of D). All data presented as mean ± SEM (**G**, *n* = 3; **J**–**O**, *n* = 7). Statistical analysis: 2-way ANOVA (**J**); 1-way ANOVA (**G**); or 2-tailed unpaired *t* test (**K**, **M**, and **O**). *P < 0.05; **P < 0.01; ***P < 0.001; ****P < 0.0001. NS, not significant.

The above studies have confirmed that activation of the LC^NE^-PF^CaMKIIα^ neural pathway promotes both pain and anxiety. To further dissect the mechanism of this pathway, we investigated the distribution and function of ARs in the PF. Based on previous literature (7, 12), to investigate whether ADRA2 in the LC-PF neural pathway plays a critical functional role, we injected anterograde-tracing viruses into the LC of *Th*-cre mice, followed by multiplex immunofluorescence histochemical staining (Figure 6, A and B). Data revealed colocalization of CaMKIIα^+^ neurons with ADRA2^+^ neurons in the PF, and these double-labeled (CaMKIIα^+^ADRA2^+^) neurons showed close apposition to anterogradely labeled NEergic fibers/terminals originating from the LC (Figure 6, C and D). Meanwhile, we performed semiquantitative Western blotting analysis of ADRA2 expression in the PF of Sham and SNI mice. Results showed a progressive increase in ADRA2 protein levels in the SNI group during chronic pain progression (Figure 6, E–G), suggesting potential involvement of ADRA2 in pain modulation. These results confirm that LC^NE^ projection activates ADRA2 on PF^CaMKIIα^ neurons.

To investigate the role of ADRA2 in the PF during chronic pain, the ADRA2 antagonist yohimbine was administered via a cannula inserted into the PF of SNI mice and behavioral assessments were conducted (Figure 6, H and I). Results showed that yohimbine had no significant effect on pain thresholds in naive mice, indicating no interference with physiological nociception. However, in SNI mice, yohimbine significantly reversed mechanical allodynia and thermal hyperalgesia (Figure 6, J and K), confirming its analgesic efficacy. In anxiety-related tests, yohimbine-treated mice exhibited increased time spent in the open arms of the EPM compared with saline controls, while no significant differences were observed in total distance or center zone exploration time in the OFT (Figure 6, L–O), suggesting selective alleviation of anxiety-like behaviors.

**Figure 7:**
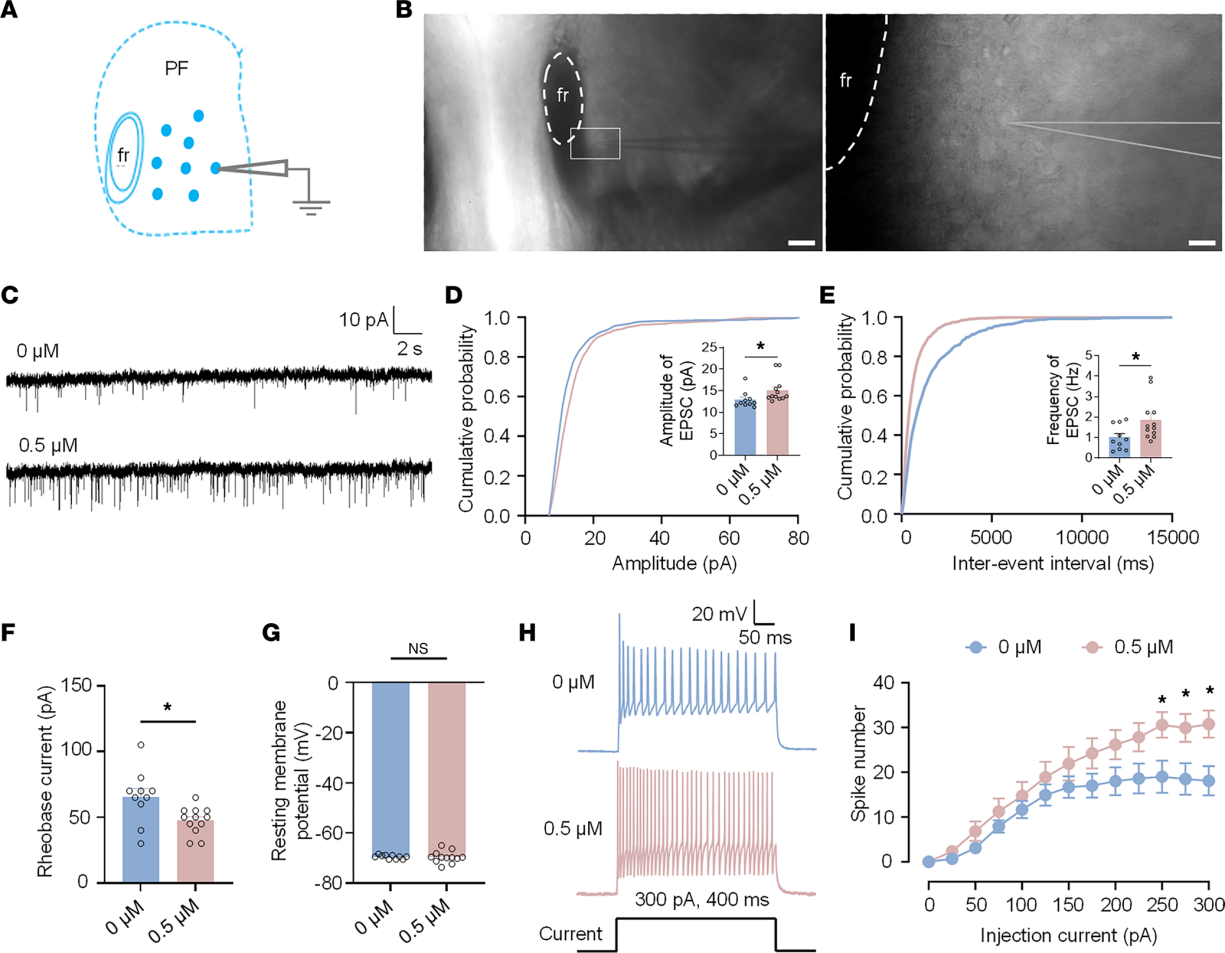
Yohimbine increases the intrinsic excitability of PF neurons. (**A**) Schematic of whole-cell patch-clamp recordings in PF neurons. (**B**) Bright-field views of clamped PF neurons. Scale bars: 40 μm (left) and 5 μm (right). (**C**) Representative sEPSCs (holding potential: –70 mV). (**D** and **E**) Amplitude and frequency of sEPSCs: statistics and cumulative curves. (**F**) Rheobase of PF neurons. (**G**) Resting membrane potential. (**H**) Action potential firing (40 ms, 300 pA current step). (**I**) Spike numbers evoked by depolarizing currents (400 ms, 25 pA increments). All data presented as mean ± SEM (**D**–**I**, *n* = 10 or *n* = 12). Statistical analysis: 2-way ANOVA (**I**); 2-tailed unpaired *t* test (**D**–**G**). *P < 0.05. NS, not significant.

To elucidate the mechanism, whole-cell patch-clamp recordings of PF neurons in SNI mice (Figure 7, A and B) revealed that ADRA2 blockade increased neuronal firing frequency and enhanced the frequency of spontaneous excitatory postsynaptic currents (sEPSCs), demonstrating that ADRA2 inhibits PF neuron intrinsic excitability to mediate pronociceptive effects (Figure 7, C–I). These findings align with previous reports of ADRA2-mediated presynaptic inhibition (15). Collectively, this study confirmed through pharmacological interventions and electrophysiological evidence that the NEergic projection from the LC to the PF produces pronociceptive effects by activating ADRA2 expressed on CaMKIIα^+^ neurons within the PF, which subsequently inhibits these CaMKIIα^+^ neurons.

## Discussion

Previous studies have demonstrated that the PF receives inputs from emotion-related brain regions, including the prefrontal cortex (PFC), primary somatosensory cortex (S1), paraventricular hypothalamic nucleus (PVN), lateral hypothalamic area (LHA), and central amygdala nucleus (CeA) (16–19), and integrates somatosensory with limbic system information through the cortico-thalamo-striatal pathway (20). The PF–anterior cingulate cortex (ACC) and CeA-PF–secondary somatosensory cortex (S2) pathways have been confirmed to mediate negative regulation of depressive-like behaviors and pain (6, 19). The results of a previous study indicate that a neural network formed by pain-related S1 neurons, PF neurons, and ACC neurons regulates pain processing and acute pain–induced anxiety-like behaviors (4). These findings collectively highlight the critical role of PF in the affective component of pain. In the present study, chemogenetic inhibition of CaMKIIα^+^ neurons in the PF of naive mice induced hyperalgesia and anxiety-like behaviors, while activation in SNI mice significantly alleviated pain and improved anxiety-like behaviors. Additionally, under chronic pain conditions, the PF serves as an information integration hub that receives inputs from the S1 (pain discrimination), amygdala (affective evaluation), and PFC (cognitive modulation), establishing a dynamic balance in pain and affective encoding (19). However, the upstream integrative mechanisms regulating PF neuronal activity during chronic pain progression remain to be elucidated.

The LC participates in pain perception, emotional regulation, and cognitive integration through its extensive ascending and descending projection networks (21). The LC^NE^ neurons are not only closely associated with thalamocortical sensory information transmission (22), but also regulate the pathophysiological mechanisms of depression and anxiety via the PFC (23, 24). Activation of the LC-PFC neural pathway induces pain, aversive emotions, and anxiety-like behaviors (10). Additionally, inhibiting the LC–basolateral amygdala nucleus (BLA) neural pathway eliminates anxiety and enhanced fear learning in rats with long-term pain, while activating this pathway induces anxiety-like behaviors and enhances aversive learning/memory indices (22). These results parallel our findings that activating the LC^NE^-PF^CaMKIIα^ neural pathway promotes pain sensitization and anxiety-like behaviors in mice, indicating LC’s critical regulatory role in anxiety. Notably, neither inhibition in naive mice nor activation in SNI mice of the LC^NE^-PF^CaMKIIα^ neural pathway significantly affected pain thresholds or anxiety-like behaviors. Firstly, the LC does not regulate basal pain threshold maintenance but provides feedback inhibition specifically during sustained pain conditions (25). Secondly, the pain and anxiety induced by SNI have plateaued at maximal levels and cannot be further exacerbated by additional activation. Recent studies demonstrate that activating the LC^NE^-SDH^CaMKIIα^ neural pathway alleviates postoperative pain (26), highlighting functional divergence between LC ascending and descending pathways. Indeed, previous studies have demonstrated that fusiform cells in the dorsal LC preferentially project to the neocortex and hippocampus, mediating higher cognitive functions (e.g., planning and decision-making), while multipolar neurons in the ventral LC predominantly innervate the spinal cord, cerebellum, and hypothalamus, mediating immediate motor responses (27–29). Our study found that 70.1% of PF-projecting LC neurons originate from the dorsal subdivision and participate in pain and anxiety regulation, further substantiating the functional heterogeneity of the LC.

A previous study has demonstrated that intracerebroventricular NE administration exerts bidirectional modulation on PF nociceptive neurons: transient inhibition (latency 2–5 minutes) during the early phase of administration, followed by sustained potentiation (30). This regulatory pattern suggests the LC-NE system suppresses spinal nociceptive transmission via descending fibers while enhancing PF pain-signal integration through ascending projections. Pharmacological blockade using phenoxybenzamine (α-adrenergic pan-antagonist) completely reversed this bidirectional effect (7), confirming LC^NE^-PF^CaMKIIα^ neural pathway–mediated pain regulation depends on ascending-descending fiber connection balance, i.e., early dominance of descending inhibition transitioning to ascending facilitation. Chemogenetic activation of the LC^NE^-PF^CaMKIIα^ neural pathway in the present study reduced mechanical pain thresholds and shortened thermal pain latencies in mice, consistent with its pronociceptive role. Electrophysiological recordings demonstrated that yohimbine elevated the action potential firing frequency of CaMKIIα^+^ neurons in the PF, suggesting that NE suppresses PF neuronal excitability through an ADRA2-mediated inhibitory mechanism, which consequently potentiates nociceptive signal transmission. Intriguingly, despite the functional opposition between the two pathways, ADRA2s in the SDH similarly inhibit CaMKIIα^+^ pain-related neurons (26). The specificity of this ADRA2-mediated inhibition in CaMKIIα^+^ neurons warrants further investigation.

Additionally, in mechanical nociceptive behavioral testing, inhibition of the LC^NE^-PF^CaMKIIα^ neural pathway significantly alleviated mechanical hypersensitivity in SNI model mice within 7 days after surgery, although the analgesic effects progressively diminished with pathological progression, suggesting that this pathway likely mediates analgesic functions specifically during the acute phase (<7 days) of neuropathic pain (31). Notably, gabapentin (an anticonvulsant agent) alleviates early neuropathic pain through dual mechanisms — suppressing presynaptic γ-aminobutyric acid release and inducing astrocytic glutamate secretion in the LC, thereby enhancing neuronal activity to activate descending NEergic inhibition (32). This pharmacodynamic profile complements our findings of LC-mediated early analgesia via ascending/descending pathways. Furthermore, a previous study has demonstrated stable electrophysiological activity in LC neurons during the first 7 days of chronic constriction injury (CCI), maintained through counterbalanced excitatory inputs from the paragigantocellular nucleus and inhibitory afferents from the dorsal raphe nucleus (33). We hypothesize that analogous homeostatic regulation in SNI model might exert therapeutic inhibition, and achieve analgesia through this balance disruption.

The present study also showed that in the hot plate test conducted 4 weeks after SNI surgery, thermal hyperalgesia was alleviated in mice. This improvement may be attributed to distinctions between mechanosensory and thermosensory pathways. Mechanical and thermal nociception are encoded by distinct spinal neuronal populations (34); mechanical pain signals are transmitted via Aδ and C-fibers (thermo-insensitive or sensitive), whereas thermal pain primarily relies on slow C-fiber conduction (35). These results suggest the LC^NE^-PF^CaMKIIα^ neural pathway may exhibit heightened sensitivity to slow C-fiber–mediated nociception. Secondly, functional heterogeneity exists among LC subpopulations. Dorsal LC neurons predominantly project to the PF, while ventral subpopulations partially mediate thermal pain processing (36). Finally, technical limitations of detection tools must be considered. The minimal threshold (0.008 g) of von Frey filaments may lack sufficient sensitivity to capture late-stage mechanical allodynia induced by subtle stimuli in chronic pain states. Additionally, this study observed significantly reduced pain thresholds in the contralateral limbs of SNI model mice, suggesting central sensitization-mediated mirror-image pain (37). Mechanism analysis revealed that the chemokine C-X3-C motif chemokine ligand 1 (CX3CL1) in the ACC enhances descending modulatory signals by activating PF^CaMKIIα^ neurons, potentially promoting bilateral nociceptive transmission (38). A previous study corroborates that selective activation of ipsilateral LC NEergic neurons alleviates mechanical allodynia in the CCI model mice, while bilateral inhibition improves pain-related depressive behaviors (39). In the present study, the synchronized bilateral pain modulation by the LC^NE^-PF^CaMKIIα^ neural pathway might involve PF neuron–mediated integration of bilateral sensory inputs via thalamocortical circuitry. However, the functional consequences of bilateral pathway inhibition require further validation.

The present results, combined with previous evidence, demonstrate that the NE system in the LC regulates stress-related negative emotional behaviors through complex molecular mechanisms, involving postsynaptic receptors (e.g., ADRA2) and anatomical projection targets (19, 21). Notably, the PF has been identified as the core downstream target mediating LC-driven pain and anxiety, providing a critical entry point for dissecting neural circuit crosstalk mechanisms between chronic pain and emotional disorders. The present study confirmed that neurons in the dorsal LC project to the PF, and the LC^NE^-PF^CaMKIIα^ neural pathway mediates pain and pain-induced anxiety-like behaviors. Furthermore, it revealed temporally distinct therapeutic effects of this pathway: potent analgesia during the acute pain phase (<7 days) and effective anxiety alleviation in chronic stages.

In summary, the present study has revealed the role of the LC^NE^-PF^CaMKIIα^ neural pathway in chronic pain and anxiety-like behaviors in mice, which may deepen our understanding of pain-anxiety comorbidity. Its pronociceptive mechanisms will promote translational applications of targeted neuromodulation strategies, such as developing ADRA2 allosteric modulators or combined chemogenetic-pharmacological intervention regimens, to achieve synergistic alleviation of pain and anxiety symptoms.

## Methods

### Sex as a biological variable.

Our study exclusively used male mice; it remains unknown whether the findings are relevant for female mice.

### Animals.

In this study, adult male C57BL/6J (Experimental Animal Center of the Fourth Military Medical University), *Th*-cre, and *Camk2a*-cre mice (both The Jackson Laboratory) were used. Animals were housed in a quiet, ventilated facility with controlled temperature (22°C–24°C), humidity (40%–60%), and a 12-hour light/dark cycle, with ad libitum access to food and water.

To investigate the distribution of Fos^+^ neurons in the LC, 12 healthy male C57BL/6J mice were divided into Sham and SNI groups (*n* = 6/group).

For semiquantitative analysis of Th protein in LC, 24 mice were divided into Sham, SNI 7-day, 21-day, and 35-day groups (*n* = 6/group). Identical cohorts were used for ADRA2 quantification in the PF.

To evaluate SNI-induced changes in nociceptive thresholds and anxiety-like behaviors, 14 mice were allocated to Sham and SNI groups (*n* = 7/group).

To investigate the effects of activating or inhibiting CaMKIIα^+^ neurons in the PF on pain thresholds and anxiety-like behaviors in naive and SNI mice, mice were divided into naive and SNI groups (21 mice per group). The naive group was subdivided into Control, hM4Di, and hM3Dq groups (*n* = 7 per subgroup), and the SNI group followed the same subgrouping. For studying the LC^NE^-PF^CaMKIIα^ neural pathway modulation, the same grouping strategy was applied to both naive and SNI groups.

To evaluate yohimbine’s effects on nociception and anxiety-like behaviors, 14 mice were allocated to saline control and yohimbine treatment groups (*n* = 7/group).

### SNI.

The SNI model was used to establish chronic neuropathic pain. Mice were anesthetized with 2% sodium pentobarbital (40 mg/kg, i.p.), followed by surgical exposure of the right sciatic nerve and its branches. The common peroneal and tibial nerves were ligated and transected. Sham mice underwent nerve exposure without ligation/transection, with other procedures identical.

### Stereotaxic injection.

Mice were anesthetized with 2% sodium pentobarbital (40 mg/kg, i.p.), and fixed in a stereotaxic apparatus (RWD), with the skull plane fully exposed and corrected. The following stereotaxic coordinates used were relative to The Mouse Brain (Paxinos & Franklin 4th ed.): PF, –2.35 mm posterior to Bregma, 0.68 mm lateral to midline, –3.58 mm ventral to brain surface; LC, –5.42 mm posterior to Bregma, 0.88 mm lateral to midline, –4.00 mm ventral to brain surface. A hole was drilled at target sites. Viruses/reagents were delivered via a glass microtube-coupled 1 μL microsyringe (Hamilton) at 20 nL/min. After the injection was completed, the needle was left in place for 10 minutes before removal.

To observe LC-to-PF projections, the retrograde tracer FG (30 nL; Biotium) was injected into the PF of C57BL/6J mice, followed by perfusion 7–10 days later. For retrograde trans-synaptic tracing, helper viruses rAAV-EF1α-DIO-RVG (150 nL; PT-0023, BrainVTA) and rAAV-EF1α-DIO-H2B-EGFP-T2A-TVA (100 nL; PT-0021, BrainVTA) were coinjected into the PF of *Camk2a*-cre mice. After 3 weeks, RV-EnvA-ΔG-DsRed (100 nL; R01002, BrainVTA) was injected into the same PF coordinates, with perfusion performed 7 days after injection. In anterograde tracing, rAAV2/9-EF1α-DIO-Ypet-2A-mGFP (150 nL; PT-0593, BrainVTA) was delivered to the LC of *Th*-cre mice, followed by perfusion after 3 weeks.

In the fiber photometry experiment, the virus rAAV2/9-hSyn-GCaMP6m (150 nL; PT-0148, BrainVTA) was injected into the PF of C57BL/6J mice, followed by fiber optic cannula (Inper) implantation above the PF.

In experiments involving chemogenetic manipulation of the PF, the virus rAAV2/9-hSyn-hM3Dq-EGFP (150 nL; PT-0152, BrainVTA), rAAV2/9-hSyn-hM4Di-EGFP (150 nL; PT-0153, BrainVTA), or control virus rAAV2/9-hSyn-EGFP (150 nL; PT-0100, BrainVTA) was injected into the PF of C57BL/6J mice. Behavioral assessments were performed 3 weeks after injection.

In experiments investigating chemogenetic modulation of the LC^NE^-PF^CaMKIIα^ neural pathway, the retrograde tracer rAAV2/R-hSyn-Cre-mCherry (150 nL; PT-0407, BrainVTA) was injected into the PF of C57BL/6J mice, and rAAV2/9-TH-DIO-hM3Dq-EGFP (150 nL; PT-6885, BrainVTA), rAAV2/9-TH-DIO-hM4Di-EGFP (150 nL; PT-6886, BrainVTA), or control virus rAAV2/9-TH-DIO-EGFP (150 nL; PT-2048, BrainVTA) was injected into the LC. Behavioral tests were conducted 3 weeks after viral delivery.

### Drug.

CNO (Abcam) was administered i.p. (1.5 mg/kg); yohimbine (TargetMol; 1 mg/mL) was microinjected into the PF via a catheter (Inper).

### von Frey test.

The PWT was measured by applying von Frey filaments (Stoelting) to the hind paw plantar surface. Mice were placed in white box on a wire mesh platform. Filaments (0.008–1.4 g) were vertically pressed against the paw. A positive response was defined as rapid paw lifting, withdrawal, or licking. Each filament intensity was tested 5 times, and the PWT was recorded as the lowest force eliciting 3 or more positive responses out of 5 trials.

### Hot plate test.

PWL was measured using an intelligent hot plate apparatus (Yiyan Tech). Mice were placed on the heated surface, and the timer was automatically terminated upon nociceptive responses (paw lifting, withdrawal, or licking), with the recorded latency defined as the thermal pain threshold. The mean latency from 3 trials was used for statistical analysis.

### OFT.

Anxiety-like behavior was assessed using an open field apparatus (SansBio). Mice were placed in the center of a white box (50 × 50 × 45 cm) for a 15-minute free exploration session. Behavior was recorded via an overhead camera. The box was thoroughly cleaned with 70% ethanol between trials. Total distance traveled and percentage of time spent in the central zone were analyzed from video recordings.

### EPM test.

The EPM apparatus (SansBio) consisted of a central platform (5 × 5 cm) and 2 opposing open arms (60 × 25 × 5 cm) elevated 50 cm above the ground. Mice were placed in the center area of the maze, with its head facing an open arm, and allowed to explore freely for 5 minutes. Total time spent in open arms and number of entries into open arms were analyzed from video recordings. The maze was cleaned with 75% ethanol between tests.

### Optical fiber and cannula implantation.

For fiber photometry, optical fibers (Inper) were implanted above the PF following viral injection. Mice were anesthetized via i.p. injection of 2% sodium pentobarbital (40 mg/kg) and secured in a stereotaxic apparatus (RWD). The fiber was implanted above the PF (coordinates: –2.35 mm posterior to bregma, 0.68 mm lateral to midline, –3.48 mm ventral from brain surface).

For yohimbine microinjection into the PF, the catheter (Inper) was stereotaxically implanted, targeting the PF. Identical experimental protocols were implemented.

### Fiber photometry recording.

Calcium signals were recorded using a multichannel fiber photometry system (Inper) to monitor GCaMP6m fluorescence in mouse brains. A 470 nm excitation light and 410 nm control light were alternately delivered (12%–15% laser power output, fiber tip intensity: 15–25 μW) at a sampling rate of 60 Hz. Data were analyzed using the manufacturer’s software (Inper Data Process).

### Patch-clamp electrophysiology.

The experimental procedures were carried out in accordance with a predefined protocol (40).

Using a fluorescence microscope, neurons exhibiting green fluorescence were selected for recordings. The neuronal membrane potential was maintained at –65 mV to facilitate the recording of sEPSCs. To induce action potentials, incrementally increasing depolarizing currents (ranging from 0 to 200 pA, with a duration of 30 ms) were injected into neurons in current-clamp mode. The minimum current required to evoke an action potential was recorded as the rheobase of the neuron. Subsequently, gradient depolarization stimuli were administered with step increments of 10 pA for 400 ms to induce the generation of consecutive action potentials. To investigate the effects of CNO on neuronal excitability, chemogenetic viruses were injected into the PF. At 3–4 weeks after injection, coronal PF-containing slices were prepared. After a 4-minute baseline recording of resting membrane potential, 10 μM CNO was added to artificial cerebrospinal fluid (ACSF), with neuronal activity recorded 5 minutes later to assess CNO effects. In the inhibitory group, the neuronal membrane potential was held at –45 mV to induce a depolarizing current. The resistance of the electrode tips varied between 2 and 7 MΩ. Data were acquired using an EPC 10 amplifier in conjunction with PatchMaster (v2 × 92, Multi Channel Systems MCS GmbH), and subsequent data analysis was conducted using Clampfit software (v11.2.2.17, Molecular Devices). To validate ADRA2-mediated inhibition of PF neuronal activity, brain tissues from SNI mice were sliced to prepare PF-containing coronal sections. Following a 4-minute baseline measurement, 0.5 μM yohimbine was administered into the ACSF, and neuronal responses were recorded 5 minutes after treatment to evaluate yohimbine’s effects.

### Immunofluorescence histochemical staining.

Mice were deeply anesthetized with 2% sodium pentobarbital (80 mg/kg, i.p.), then perfused with 0.01 mol/L phosphate-buffered saline (PBS, pH 7.4) and 4% paraformaldehyde in 0.1 mol/L phosphate buffer (pH 7.4). After perfusion, the brains were transferred to 30% sucrose solution. Coronal brain sections (30 μm) were cut using a cryostat (Leica), divided equally into 6 sets. The first set was observed under a fluorescence microscope (Olympus), the second set was used for Nissl staining, and the third/fourth sets were used for immunofluorescence histochemistry. The remaining 2 sets were used for control test of replacing specific antibodies with normal rabbit and mouse serum. For immunofluorescence histochemical staining, the sections were first incubated in 10% donkey serum blocking solution at room temperature for 30 minutes. Then, the sections were incubated in primary antibody at 4°C for 24 hours. The primary antibodies included normal rabbit serum (ab7487, Abcam), normal mouse serum (ab7486, Abcam), rabbit anti-FG (1:400; AB153I, Merck Millipore), mouse anti-Th (1:400; AB5986, Merck Millipore), rabbit anti-CaMKIIα (1:400; ab34703, Abcam), rabbit anti-Fos (1:400; ab22699, Abcam), mouse anti-Fos (1:400; ab11959, Merck Millipore), and mouse anti-ADRA2 (1:400; AWA43082, Abwiz). Then, the sections were incubated in secondary antibody at room temperature for 4 hours. The secondary antibodies included Alexa Fluor 594–anti-rabbit (1:400; A21207, Invitrogen), Alexa Fluor 488–anti-mouse (1:400; A21202, Invitrogen), and Alexa Fluor 647–anti-mouse (1:400; A31573, Invitrogen). Finally, the sections were incubated in DAPI solution (1:1000; D9564, Sigma-Aldrich) for 5 minutes, mounted onto glass slides using antifade sealing medium, and imaged under a laser scanning confocal microscope (FV1000, Olympus).

### Nissl staining.

The first set section was mounted on gelatin-coated slides and incubated in 70% alcohol (Sinopharm) at 37°C overnight. The slides were then immersed in Nissl staining solution (Sinopharm) for 30–60 minutes. Subsequently, the slides were sequentially immersed in 70%, 80%, and 90% alcohol for 2 minutes each, followed by 95% and 100% alcohol for 10 minutes each. The sections were soaked in xylene (Sinopharm) for 30 minutes, transferred to fresh xylene for 2 hours, and then removed to dry. Finally, the slides were sealed with neutral resin (DPX, Sigma-Aldrich) and air-dried.

### Western blotting.

Mice were deeply anesthetized with 2% sodium pentobarbital (80 mg/kg, i.p.), and the entire brains were swiftly extracted. The PF and LC were extracted and uniformly mixed using an automated rapid sample homogenizer (Jingxin Industry) in sodium dodecyl sulfate (SDS) sample buffer. Subsequently, all the samples were heated in a water bath at 100°C for 10 minutes, loaded onto gels, and separated via electrophoresis in 10% SDS-polyacrylamide gels utilizing standard Laemmli solutions (Bio-Rad). The proteins were then blotted onto polyvinylidene difluoride membranes (Millipore). Next, the membranes were immersed in a blocking solution for 1 hour, followed by overnight incubation with primary antibodies rabbit anti-Th (1:1000; AB152, Merck Millipore), mouse anti-ADRA2A (1:400; AWA43082, Abwiz), or mouse anti–β-actin (1:5000; A1978, Sigma-Aldrich). After that, we applied horseradish peroxidase–conjugated (HRP-conjugated) secondary antibodies to detect primary antibodies, specifically anti-rabbit (1:5000; ZB-2301, ZSGB-BIO) or anti-mouse (1:5000; ZB-2305, ZSGB-BIO). Visualization of all reactions was achieved through the enhanced chemiluminescence (ECL) detection technique, and the intensities of the protein bands were quantified using Labworks Software (Ultra-Violet Products).

### Statistics.

All data are presented as mean ± SEM. The normality of each data set was initially assessed using the Shapiro-Wilk test, and those that conformed to a normal distribution were subjected to parametric analyses. We used unpaired 2-tailed *t* tests to compare the means between 2 groups. Non–normally distributed data sets were analyzed using the Mann-Whitney rank-sum test. Comparisons across 3 or more groups were performed using 1-way ANOVA or 2-way ANOVA, followed by Holm-Šídák post hoc test. All statistical analyses were performed using Prism 9.0 software (GraphPad). A significance threshold of *P* less than 0.05 was applied.

### Study approval.

All experimental protocols were approved by the Institutional Animal Care and Use Committee of the Fourth Military Medical University (Xi’an, China).

### Data availability.

All data needed to evaluate the conclusions in the paper are present in the paper and/or the supplement. Values for all data points shown in graphs and behind any reported means are available in the Supporting Data Values file.

## Author contributions

YQL and FL conceived the project and designed the experiments. ZYL, JC, and SST performed the behavioral tests. ZYL, HMT, JL, TYZ, and DNL completed morphological staining. ZYL, JC, and JL performed the molecular experiments. LML, ZAL, and FSH performed the in vitro electrophysiological experiments. YHL performed the calcium imaging analysis. ZYL, FL, and YQL drafted the manuscript. FL, HL, and YQL edited the final version of the manuscript. All authors have read and approved the final manuscript.

## Conflict of interest

The authors have declared that no conflict of interest exists.

## Funding support

- National Natural Science Foundation of China (grants 82130034, 82471254, and 82221001).
- Brain Science and Brain-like Intelligence Technology – National Science and Technology Major Project (2021ZD0204403).
- The Fourth Military Medical University Partner Lab Cooperation and Exchange Project (2022HB002).

## Supplementary Material

Supplemental data

Unedited blot and gel images

Supporting data values

## Version 1 04/07/2026

In-Press Preview

## Version 2 05/22/2026

Electronic publication
