# Supplementary material for: Norepinephrinergic projection from locus coeruleus to parafascicular nucleus promotes pain and anxiety-like behaviors in mice
Source: JCI Insight. 2026 Apr 7;11(10):e198224. doi: 10.1172/jci.insight.198224 (PMC13232715; doi:10.1172/jci.insight.198224)
Supplement: Supplemental data [file jciinsight-11-198224-s086.pdf]

Supplemental material

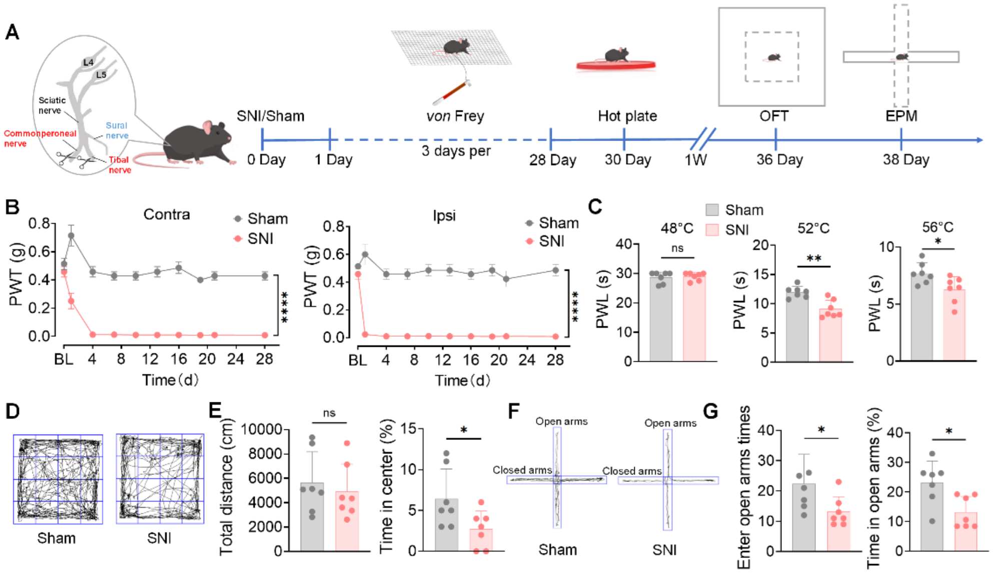

Supplemental Figure 1. Chronic pain promotes nociceptive hypersensitivity and anxiety-like behaviors in mice. (**A**) Schematic of spared nerve injury (SNI) modeling and behavioral experimental timeline in male C57BL/6J mice. (**B**) PWT (g) in ipsilateral (right) and contralateral (left) hind paws of Sham and SNI groups mice at postoperative days 0, 1, 4, 7, 10, 13, 16, 19, 21, and 28, respectively. (**C**) PWL (s) of Sham and SNI mice exposed to 48°C, 52°C, and 56°C hot plates. (**D**) Representative open field test (OFT) movement trajectories for Sham and SNI groups mice. (**E**) Total distance (left) and percentage time spent in the center zone (right) during OFT in Sham and SNI groups mice. (**F**) Representative elevated plus maze (EPM) movement trajectories for Sham and SNI groups mice. (**G**) Times of entries into open arms (left) and percentage time spent in open arms (right) during EPM in Sham and SNI groups mice. All data are expressed as mean ± SEM ($n = 7$). Statistical analysis: two-way ANOVA (**B**); two-tailed unpaired *t*-test (**C**, **E** and **G**). Significance levels: *$P < 0.05$, **$P < 0.01$, ***$P < 0.001$, ****$P < 0.0001$; *ns* = not significant.

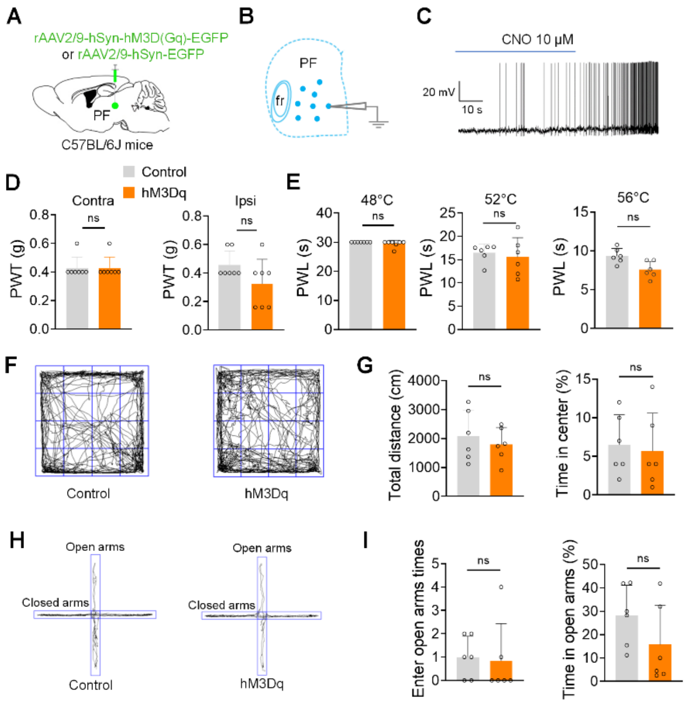

Supplemental Figure 2. Chemogenetic activation of $PF^{CaMKII\alpha}$ neurons does not alter pain thresholds or anxiety-like behaviors in naïve mice. (**A**) Schematic of experimental design. (**B**) Schematic of patch-clamp experimental design. (**C**) Representative current-clamp recordings of hM3Dq neurons treated with 10 μM CNO. (**D** and **E**) Effects of chemogenetic activation of the $PF^{CaMKII\alpha}$ neurons on mechanical (**D**) and thermal (**E**) pain thresholds in naïve mice. (**F**) Representative OFT movement trajectories for Control and hM3Dq groups mice. (**G**) Total distance (left) and percentage time in center zone (right) during OFT in Control and hM3Dq groups mice. (**H**) Representative EPM movement trajectories for Control and hM3Dq groups mice. (**I**) Times of open arm entries (left) and percentage time in open arms (right) during EPM in Control and hM3Dq groups mice. All data are expressed as mean ± SEM (n = 7). Statistical analysis: two-tailed unpaired t-test; *ns* = not significant.

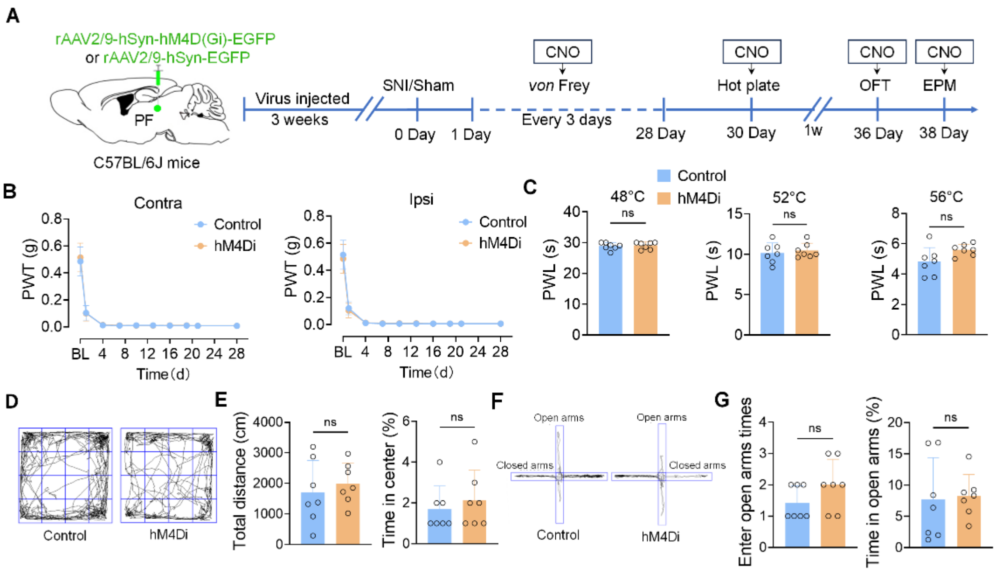

Supplemental Figure 3. Inhibition of $\mathrm{PF}^{\mathrm{CaMKII}\alpha}$ neurons alleviates pain and anxiety-like behaviors in SNI mice. (**A**) Schematic of experimental design. (**B**) PWT (g) in contralateral (left) and ipsilateral (right) hind paws of Control and hM4Di groups at postoperative days 0, 1, 4, 7, 10, 13, 16, 19, 21, and 28, respectively. (**C**) PWL (s) of Control and hM4Di groups mice under 48°C, 52°C, and 56°C hot plate stimuli. (**D**) Representative OFT movement trajectories for Control and hM4Di groups mice. (**E**) Total distance (left) and percentage time in center zone (right) during OFT in Control and hM4Di groups mice. (**F**) Representative EPM movement trajectories for Control and hM4Di groups mice. (**G**) Times of open arm entries (left) and percentage time in open arms (right) during EPM in Control and hM4Di groups mice. All data are expressed as mean ± SEM (n = 7). Statistical analysis: two-way ANOVA (**B**); two-tailed unpaired t-test (**C**, **E** and **G**). Significance levels: *ns* = not significant.

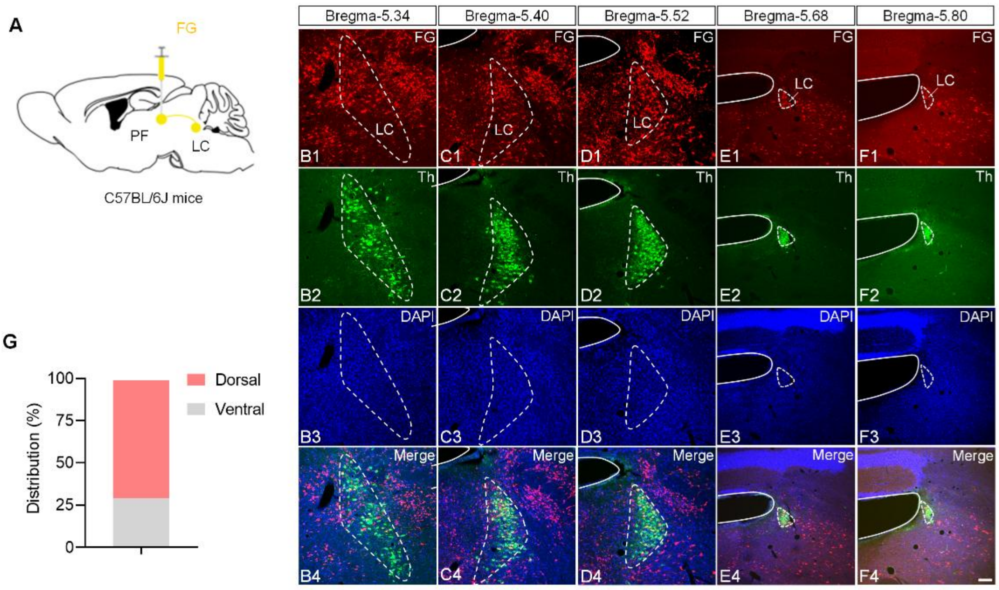

31 **Supplemental Figure 4. Distribution patterns of FG$^+$ neurons in the LC. (A) Schematic of FG injection into**
32 **the PF.** (**B1-F4**) Distribution of FG$^+$ neurons (red) across coronal planes of the LC and their co-localization with
33 Th$^+$ neurons (green). Blue, DAPI. Scale bars: 120 μm. (**G**) Distribution of FG$^+$ neurons in the ventral and dorsal of
34 LC.

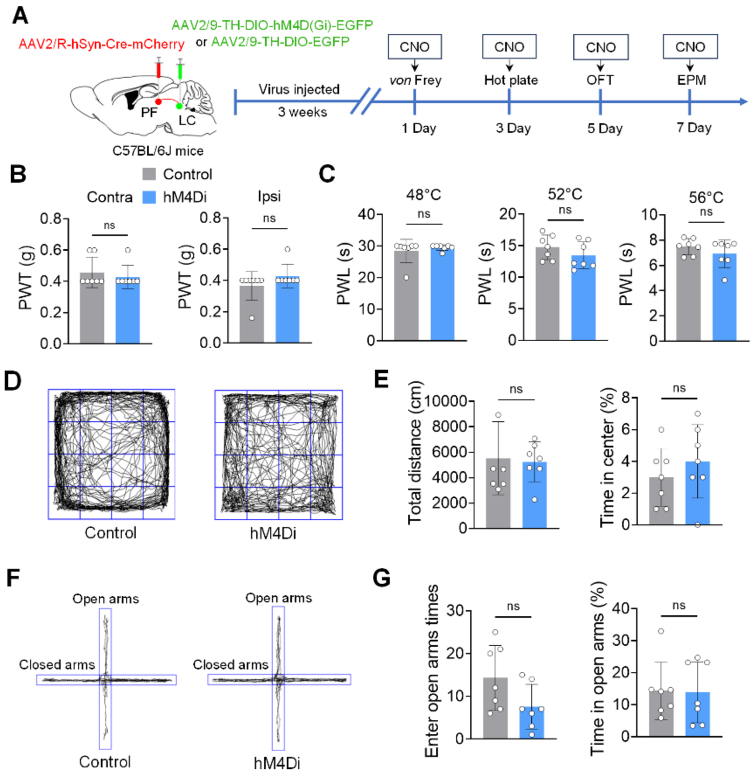

Supplemental Figure 5. Inhibition of the LC^{NE}-PF$^{CaMKII\alpha}$ neural pathway promotes nociceptive hypersensitivity and induces anxiety-like behaviors. (**A**) Schematic of pathway inhibition experimental design. (**B** and **C**) Effects of chemogenetic inhibition of the LC^{NE}-PF$^{CaMKII\alpha}$ neural pathway on mechanical (**B**) and thermal (**C**) pain thresholds in naïve mice. (**D**) Representative OFT movement trajectories for Control and hM4Di groups mice. (**E**) Total distance (left) and percentage time in center zone (right) during OFT in Control and hM4Di groups mice. (**F**) Representative EPM movement trajectories for Control and hM4Di groups mice. (**G**) Times of open arm entries (left) and percentage time in open arms (right) during EPM in Control and hM4Di groups mice. All data presented as mean ± SEM (n = 7). Statistical analysis: two-tailed unpaired t-test. Significance levels: *ns* = not significant.

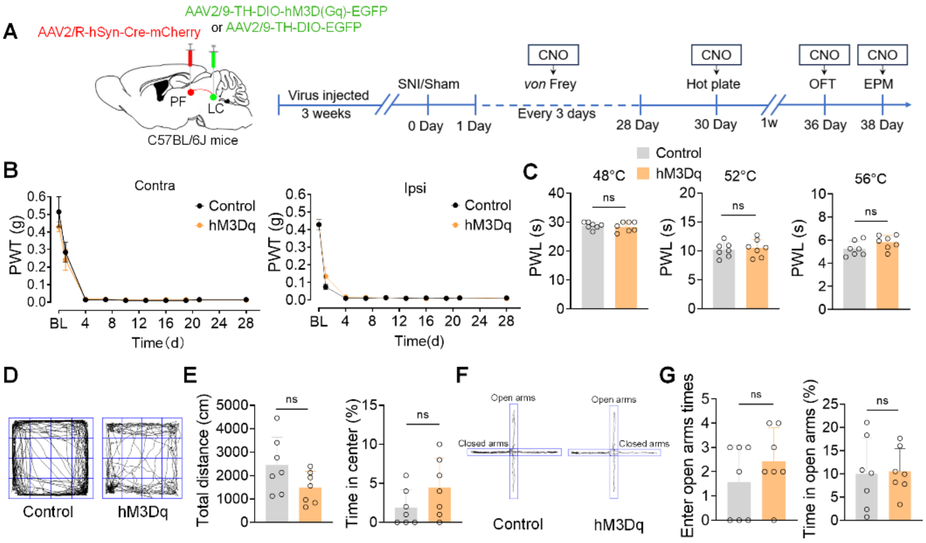

Supplemental Figure 6. Activation of the LC^{NE}-PF$^{CaMKII\alpha}$ neural pathway shows no significant effects on nociceptive hypersensitivity or anxiety-like behaviors in SNI mice. (**A**) Schematic of pathway activation experimental design. (**B** and **C**) Effects of chemogenetic activation of the LC^{NE}-PF$^{CaMKII\alpha}$ neural pathway on mechanical (**B**) and thermal (**C**) pain thresholds in SNI mice. (**D**) Representative OFT movement trajectories for Control and hM3Dq groups mice. (**E**) Total distance (left) and percentage time in center zone (right) during OFT in Control and hM3Dq groups mice. (**F**) Representative EPM movement trajectories for Control and hM3Dq groups mice. (**G**) Times of open arm entries (left) and percentage time in open arms (right) during OFT in Control and hM3Dq groups mice. All data presented as mean ± SEM (n = 7). Statistical analysis: two-way ANOVA (**B**); two-tailed unpaired t-test (**C**, **E** and **G**). Significance levels: *ns* = not significant.

Supplemental Table 1. Quantification and distribution of FG^+ neurons, Th^+ neurons and FG^++Th^+/FG^+ neurons in the LC.

Mouse ID	FG^+ neurons in the ventral LC	FG^+ neurons in the dorsal LC	FG^+ neurons	Th^+ neurons	FG^++Th^+ neurons	FG^++Th^+/FG^+ (%)
1	59	120	179	980	171	95.5
2	51	90	145	1032	130	89.7
3	55	178	233	984	212	91.0
mean ± SEM	55 ± 3.27	129 ± 36.52	186 ± 36.23	999 ± 23.62	171 ± 33.48	92.1 ± 2.5

Supplemental Table 2. Quantification of DsRed^{+} neurons and DsRed^{+}+Th^{+} neurons in the LC.

Mouse ID	DsRed^{+} neurons	DsRed^{+}+Th^{+} neurons	DsRed^{+}+Th^{+}/DsRed^{+} (%)
1	56	46	82.14
2	14	20	70
3	34	44	77.27
mean ± SEM	35 ± 17.15	37 ± 11.81	76.47 ± 4.98

Supplemental Table 3. Quantification of Fos^+ neurons in the LC of Sham and SNI Mice.

Mouse ID	Sham group	SNI group
1	196	640
2	180	645
3	268	666
4	219	592
5	80	540
6	147	618
mean ± SEM	182 ± 58.58	617 ± 41.39

Supplemental Table 4. Expression and co-localization of Fos^+ and Th^+ neurons in the LC.

Mouse ID	Fos^+ neurons	Th^+ neurons	Fos^++Th^+ neurons	Fos^++Th^+/Fos^+ (%)
1	640	885	463	72.31
2	645	894	466	72.14
3	666	936	474	71.15
4	592	843	416	70.22
5	540	807	362	66.91
6	618	966	396	63.97
mean ± SEM	617 ± 41.36	872 ± 68.07	392 ± 22.29	67 ± 2.55
