# Supplementary material for: Norepinephrinergic projection from locus coeruleus to parafascicular nucleus promotes pain and anxiety-like behaviors in mice
Source: JCI Insight. 2026 Apr 7;11(10):e198224. doi: 10.1172/jci.insight.198224 (PMC13232715; doi:10.1172/jci.insight.198224)
Supplement: Unedited blot and gel images [file jciinsight-11-198224-s087.pdf]

Full unedited gel for Figure 5B.

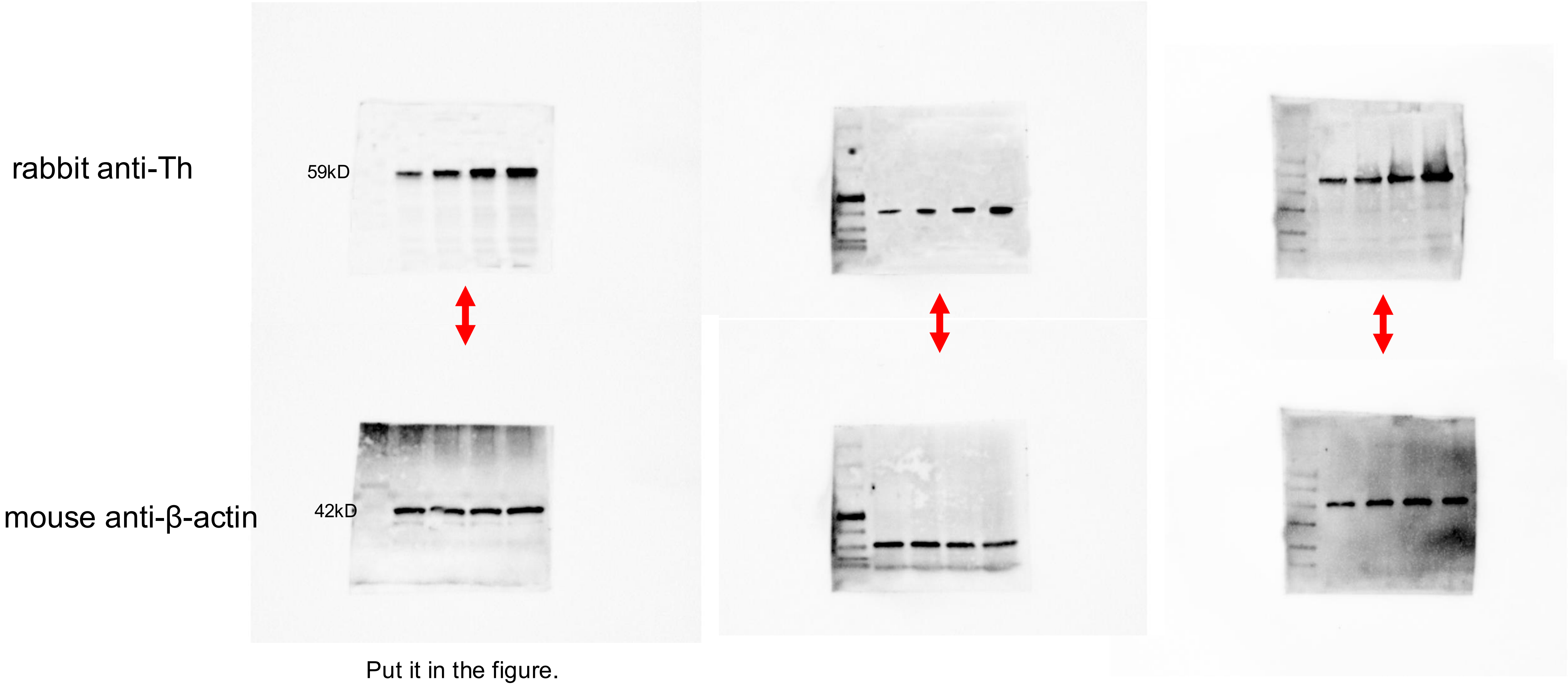

Put it in the figure.

Full unedited gel for Figure 6F.

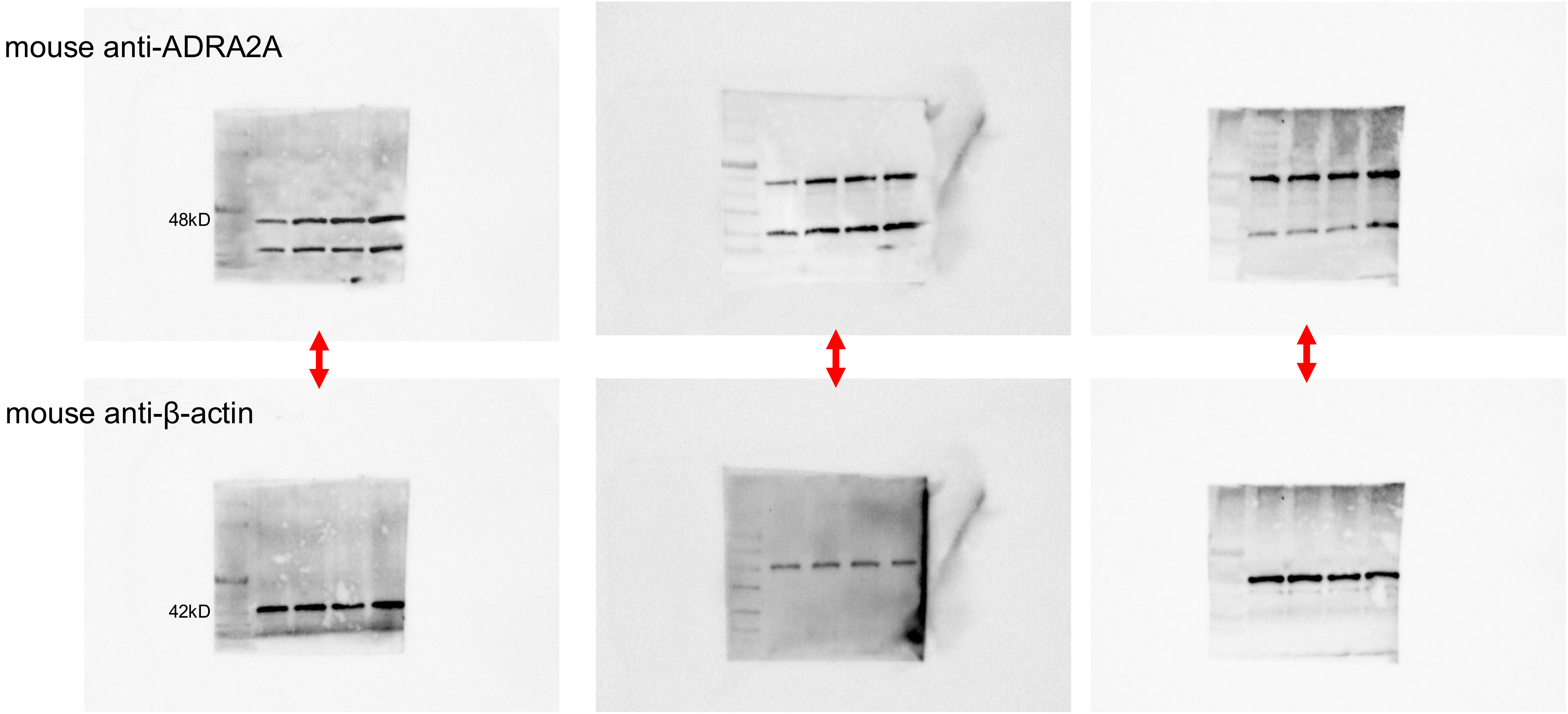

Put it in the figure.
